# Single-cell transcriptomic profiling of microvascular endothelial cell heterogeneity in congenital diaphragmatic hernia

**DOI:** 10.1038/s41598-023-37050-y

**Published:** 2023-06-17

**Authors:** Jason O. Robertson, Peter Bazeley, Serpil C. Erzurum, Kewal Asosingh

**Affiliations:** 1 Department of Pediatric Surgery, Digestive Disease and Surgery Institute, Cleveland Clinic Children’s, 9500 Euclid Avenue/A10, Cleveland, OH 44195 USA; 2 Department of Quantitative Health Sciences, Cleveland Clinic Lerner Research Institute, Cleveland, 44195 USA; 3 Department of Inflammation and Immunity, Cleveland Clinic Lerner Research Institute, Cleveland, 44195 USA

**Keywords:** Angiogenesis, Disease model, Intrauterine growth, Organogenesis

## Abstract

Congenital diaphragmatic hernia (CDH) is a neonatal anomaly that includes pulmonary hypoplasia and hypertension. We hypothesized that microvascular endothelial cell (EC) heterogeneity is different in CDH lungs and related to lung underdevelopment and remodeling. To test this, we evaluated rat fetuses at E21.5 in a nitrofen model of CDH to compare lung transcriptomes among healthy controls (2HC), nitrofen-exposed controls (NC) and nitrofen-exposed subjects with CDH. Single-cell RNA sequencing with unbiased clustering revealed 3 distinct microvascular EC clusters: a general population (mvEC), a proliferative population and a population high in hemoglobin. Only the CDH mvEC cluster had a distinct inflammatory transcriptomic signature as compared to the 2HC and NC endothelial cells, e.g. greater activation and adhesion of inflammatory cells and production of reactive oxygen species. Furthermore, CDH mvECs had downregulated *Ca4*, *Apln* and *Ednrb* gene expression. Those genes are markers for ECs important to lung development, gas exchange and alveolar repair (mvCa4+). mvCa4+ ECs were reduced in CDH (2HC [22.6%], NC [13.1%] and CDH [5.3%], *p* < 0.0001). Overall, these findings identify transcriptionally distinct microvascular endothelial cell clusters in CDH, including the distinctly inflammatory mvEC cluster and the depleted group of mvCa4+ ECs, which together may contribute to pathogenesis.

## Introduction

Congenital diaphragmatic hernia (CDH) is a severe developmental anomaly that affects 1 out of every 2500 newborns. Significant pulmonary hypoplasia and pulmonary hypertension (PH) complicate the disease and result in high morbidity and mortality^1^. Lungs in these infants are characterized by smaller, fewer and more primitive alveoli, thickening of the alveolar septa in the terminal airways^2,3^, and microvascular networks that are less extensive with decreased arborization and density^4^. These changes are generally considered to be a consequence of two hits: (1) as yet unidentified underlying pathologic changes and (2) the consequences of lung compression^5–9^. The most common model of CDH involves administration of the herbicide nitrofen to pregnant rats, which results in diaphragmatic defects, pulmonary hypoplasia and pulmonary hypertension in fetuses^10–12^. The nitrofen model mirrors the two hit development of lung disease in CDH, as well as the decreased vascularity and alveolar changes^13^. 

Lung vascular development progresses most rapidly during the fetal period of lung development. The last stage of fetal lung development, the saccular stage, occurs between 26–38 weeks gestation in humans and embryonic day (E) 20 to postnatal day (P) 4 in rats^10^. In this stage, there is a tremendous expansion of the airway surface that is accompanied by a significant increase in capillary growth^10^. These developmental processes require a high level of spatial coordination and precise endothelial-epithelial crosstalk for proper completion. There is increasing evidence that endothelial cell heterogeneity in the vasculature is substantial and that these distinct functional and/or spatial subsets of cells uniquely contribute to health and disease^14–19^. The role of endothelial cell heterogeneity during fetal lung development in CDH and how derangements in different endothelial clusters contribute to the disease has not been previously studied.

Under-developed and remodeled lung and pulmonary vasculature are the central components of the most severe CDH-associated PH^20,21^. Sprouting of new capillaries at the expanding tips of the distal epithelium, a process known as distal angiogenesis^22^, is rate limiting to airway formation and particularly important during maturation of sacculi and alveoli^23^ to the point that decreased lung vascular growth during development contributes to the etiology of pulmonary hypoplasia^24,25^. Given this and the fact that the vast majority of the lung endothelium is microvascular^26,27^, a closer delineation of microvascular endothelial cells in CDH is warranted to better understand the disease pathogenesis. All studies to date have looked at individual factors or pathways and hypothesized focused connections to pulmonary hypoplasia and hypertension, but none have taken an unbiased, single cell transcriptomics approach to study developing pulmonary endothelium^12,28^.

We hypothesized that microvascular endothelial cells contribute to abnormal lung development in congenital diaphragmatic hernia. To test this, we used single cell RNA-sequencing (scRNA-seq) to characterize pulmonary endothelial cell heterogeneity and differential gene expression (DGE) patterns in E21.5 healthy, nitrofen exposed and nitrofen-induced CDH lungs. This enabled identification of multiple, transcriptionally distinct microvascular endothelial cell subclusters and determination of the impact of lung compression (i.e. the second hit).

## Results

### Phenotyping of E21.5 Fetal Lungs

**Figure 1:**
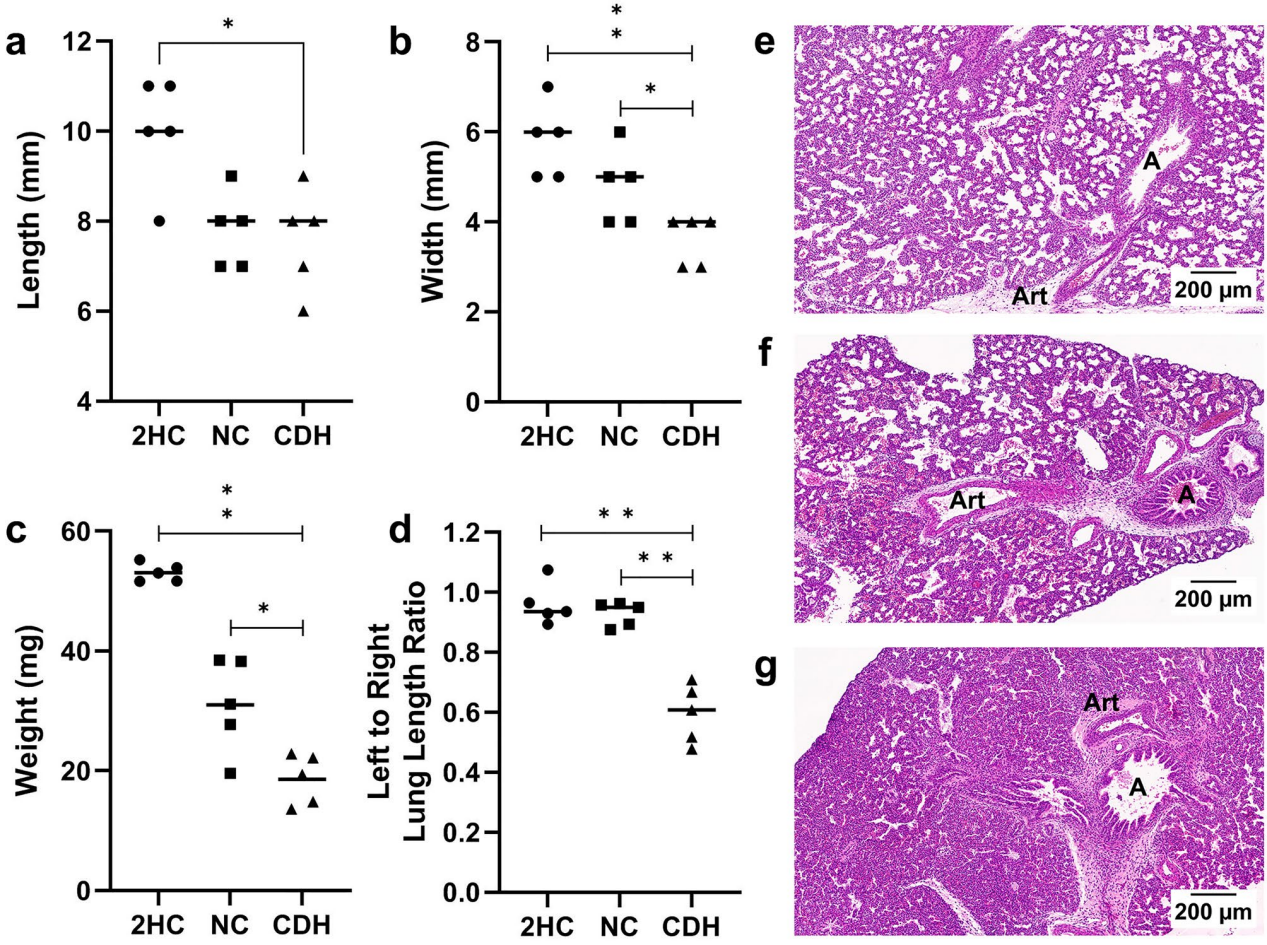
Left CDH lungs selected for scRNA-seq were significantly more hypoplastic than healthy controls (2HC) and nitrofen exposed lungs without an ipsilateral diaphragmatic defect (NC). Measurements of length (**a**), width (**b**) and weight (**c**) were decreased in a stepwise fashion with nitrofen exposure and lung compression in the NC (n = 5) and CDH (n = 5) groups, respectively. Only lungs included in the analysis are included in these graphs. Lung compression affected the lungs ipsilateral to the diaphragmatic defect causing discordance between the sizes of the left and right lungs (**d**). Hematoxylin and eosin stains show representative sections of lungs similar to those used for scRNA-seq for 2HC (**e**), NC (**f**) and CDH (**g**). These images show hypoplasia in both the NC and CDH lungs with progressive thickening of the alveolar septums, obliteration of the alveolar airspaces and thickening of the arteries. Scale bars are at 200 µm. A = airway, Art = artery.

Whole cell scRNA-seq was performed with fresh preparations of fetal lung tissue, making selection of lungs dependent on gross rather than microscopic phenotyping (Fig. 1a–d). The two measurements that were weighted most heavily in selecting lungs for analysis were the weight and the left-to-right lung length ratios. Mean (± SD) weights of lungs selected for each group were 53.1 ± 1.5 g, 31.0 ± 7.9 g, and 18.6 ± 4.2 g for the 2HC, NC and CDH groups, respectively (*p* < 0.0001) (Fig. 1c). This compared to average weights of 46.1 ± 3.5 g for unused 2HC lungs and 16.7 ± 4.3 g for the unused CDH lungs. Fewer nitrofen exposed lungs were available from our experimental rats, so only one was unused, weighing 18.8 g. Therefore, CDH lungs used for scRNA-seq were on average 35% the size of healthy controls and 60% the size of nitrofen exposed lungs without diaphragmatic defects. Lungs from the NC group were most variable in size across rats.

Lung weights can vary with size of the fetuses, so measuring the ratio of the left-to-right lung length provided the best assessment of how lung compression affected the development of the lung ipsilateral to the diaphragmatic defect. On average, selected left CDH lungs were 59.5 ± 9.7% the length of their matched right lung (Fig. 1d). Even though selected lungs were slightly heavier than unused lungs, the unused left lungs were on average 72.4 ± 6.3% the length of the right lungs, indicating that the selected lungs were impacted more by left lung compression. Left and right lungs from the 2HC and NC groups were evenly matched in terms of length.

Representative hematoxylin and eosin stains of lungs similar to those used for scRNA-seq (from the same litters of pups for 2HC and CDH) are additionally presented for 2HC (Fig. 1e), NC (Fig. 1f) and CDH (Fig. 1g) to illustrate the phenotype. Histology demonstrated progressive thickening of the alveolar septae and pulmonary arteries and obliteration of the alveolar spaces consistent with the known changes in our model. These sections showed clear changes from lung compression that were additive to the effect of nitrofen alone.

### Composition and identification of cell types in E21.5 fetal rat lungs

**Figure 2:**
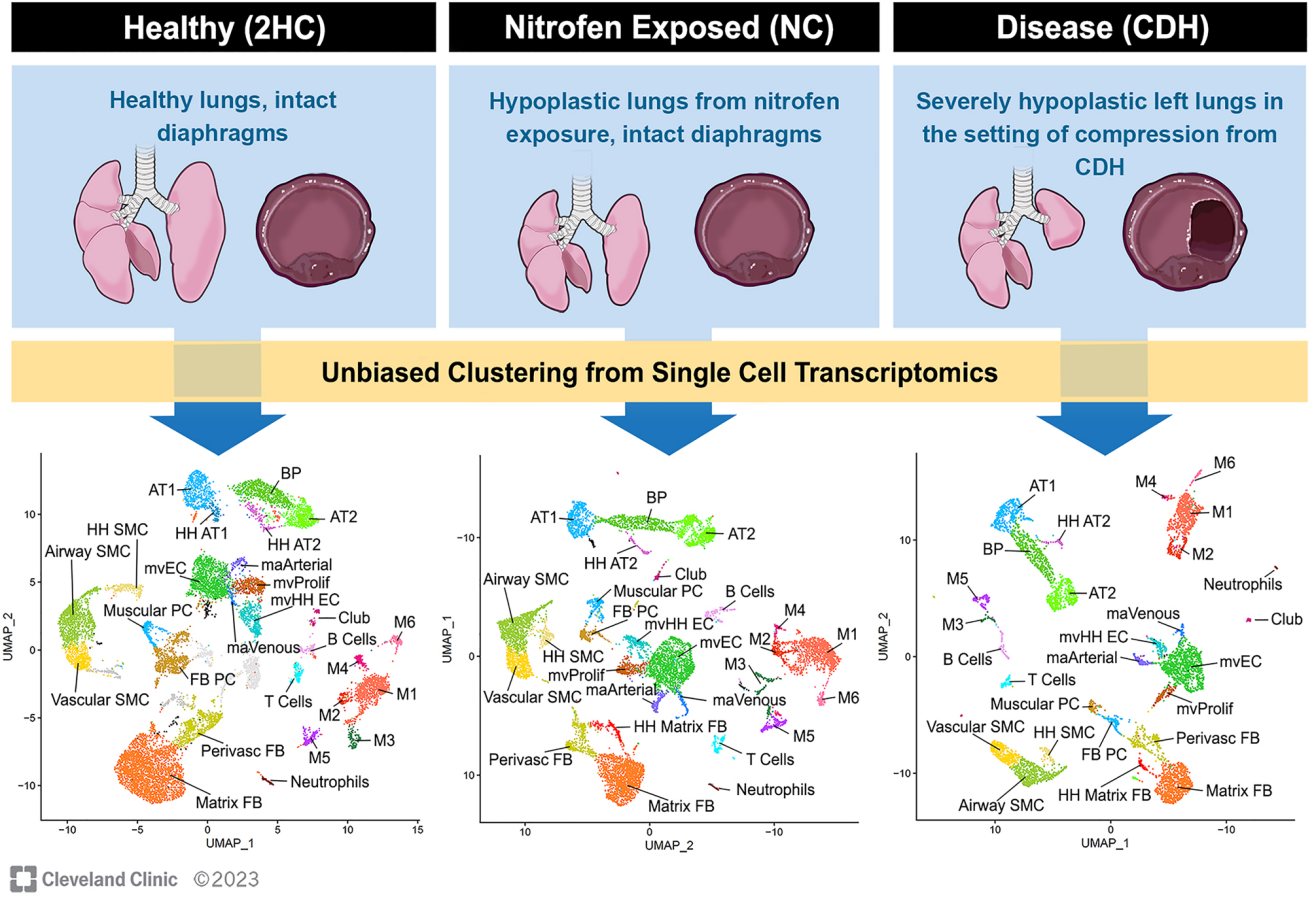
Overview of experimental groups and identification of endothelial cell clusters from whole lung single cell preparations. Left lungs were collected from three experimental groups of rat fetuses during the saccular stage of fetal lung development (E21.5) and pooled for analysis. The healthy control group (2HC, n = 10) received a sham treatment and developed with both normal diaphragms and lungs. The nitrofen control (NC, n = 5) group acquired comparatively mild bilateral lung hypoplasia following exposure to nitrofen but developed with intact diaphragms. The CDH group (n = 5) consisted of nitrofen exposed fetuses that developed with a ≥ 50% left-sided diaphragmatic defect, resulting in a “second hit” of lung compression from herniated abdominal viscera. Single cell transcriptomic profiling was performed for pooled lung specimens from each of these three groups. Major lung cell types were identified within UMAPs that were generated using an iterative, unbiased clustering strategy. This approach identified 5 separate clusters of endothelial cells, including two macrovascular cell clusters (maArterial and maVenous) and three microvascular cell clusters (mvEC, mvHH, and mvProlif). AT1 = type I alveolar cells, AT2 = type 2 alveolar cells, BP = bipotential alveolar progenitors, FB = fibroblasts, FB PC = fibroblastic pericytes, HH = high hemoglobin, PC = pericytes, Perivasc FB = perivascular fibroblasts, SMC = smooth muscle cells, M1-M6 = different populations of macrophages.

Figure 2 provides an overview of the experimental groups. Clusters for the healthy lung control group (2HC), the nitrofen exposed control group (NC), and the nitrofen-exposed CDH group (CDH) are shown (Fig. 2, lower panels). No unique clusters were apparent between groups. All clusters were identified by using known cell type selective markers following unbiased high resolution clustering (Supplemental Figs. S1, S2, and S3)^29–35^. Endothelial cells comprised 15.9%, 20.6% and 20.9% of total cells for the 2HC, NC and CDH groups, respectively. These counts included five endothelial cell subclusters: the general population of microvascular ECs (mvEC), proliferative microvascular ECs (mvProlif), microvascular ECs high in hemoglobin (mvHH), macrovascular arterial ECs (maArterial) and macrovascular venous ECs (maVenous) (Supplemental Fig. S1). Characteristics of microvascular *Ca4*^+^ endothelial cells are reported, but these cells were manually selected based on *Ca4*^+^ gene expression and do not represent a discrete cluster of ECs on our UMAPs (Supplemental Fig. S1).

Table 1 analyzes changes in the proportion of each endothelial cell subcluster as a percentage of all endothelial cells across experimental groups. These data show that the proportion of mvECs increases incrementally between 2HC and NC and again between NC and CDH (53.4% vs. 64.4% vs. 70.0%, respectively). This is the only significant difference between proportions of NC and CDH endothelial cells (*p* = 2.31E−26). However, our data are also notable in that nitrofen exposure decreases the proportion of mvProlif and mvHH clusters. These proportions decreased slightly more in the CDH group, and the differences between NC and CDH mvHH clusters narrowly missed significance (11.5% vs. 8.5%, *p* = 0.0541). Loss of these cell clusters is what allows for the increase in mvEC proportion. No significant changes were observed for the macrovascular endothelial cells.

**Table 1:** Cell counts and proportion analyses for each endothelial cell cluster as a percentage of all endothelial cells.

Cell population	2HC (total cells = 2548)	NC (total cells = 2489)	CDH (total cells = 1350)	2HC versus CDH *P *value*	2HC versus NC *P *value*	CDH versus NC *P *value*
mvEC	1361 (53.4%)	1602 (64.4%)	945 (70.0%)	1.80E−22	4.39E−14	0.0063
mvProlif	484 (19.0%)	289 (11.6%)	127 (9.4%)	7.08E−14	5.41E−12	0.5393
mvHH	445 (17.5%)	287 (11.5%)	115 (8.5%)	5.31E−13	3.46E−08	0.0541
maArterial	146 (5.7%)	168 (6.7%)	97 (7.2%)	1.0000	1.0000	1.0000
maVenous	112 (4.4%)	143 (5.7%)	66 (4.9%)	1.0000	0.433881	1.0000

CDH = congenital diaphragmatic hernia experimental group, 2HC = healthy control experimental group, maArterial = macrovascular arterial endothelial cells, maVenous = macrovascular venous endothelial cells, mvEC = main cluster of microvascular endothelial cells, mvHH = high hemoglobin microvascular endothelial cells, mvProlif = proliferative microvascular endothelial cells, NC = nitrofen exposed experimental group.

### Analysis of mvEC differential gene expression

Our analysis was focused on the microvascular ECs, particularly the mvEC population, since microvascular ECs are most relevant to alveolar development and expansion and because mvECs were the largest cluster. Within our differential gene expression (DGE) cutoff of log_2_ fold change (log_2_FC) >|1|, there were 41 genes up- and 64 genes down-regulated in CDH versus 2HC mvECs, 12 genes up- and 35 genes down-regulated in NC versus 2HC mvECs, and 0 genes up- and 4 genes down-regulated in CDH vs NC mvECs (Supplemental Excel File).

**Figure 3:**
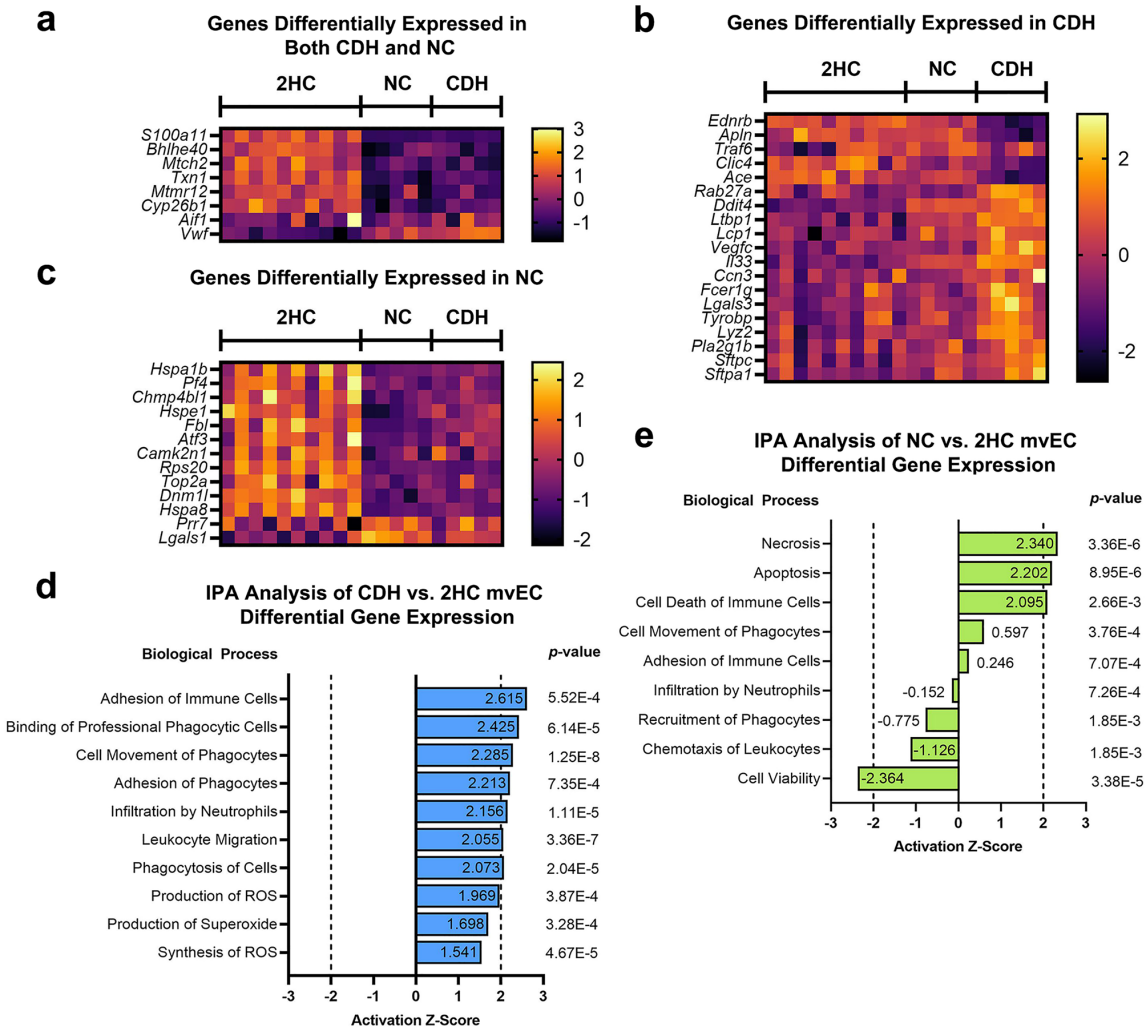
Differentially expressed genes in the general microvascular endothelial cell cluster (mvEC) predicted increased inflammation and production of reactive oxygen species in CDH lungs. Differential gene expression was determined for both CDH and NC lungs compared to healthy controls and analyzed by Ingenuity Pathway Analysis (IPA). Expression of genes attached to biological pathways detected by IPA are shown in heatmaps as differentially expressed in both CDH and NC lungs (**a**), differentially expressed in only CDH lungs (**b**) and differentially expressed in only NC lungs (**c**). Differentially expressed mvEC genes identified in the heatmaps are sorted by smallest to largest log2FC. Several biological processes related to inflammation and free radical production were predicted by IPA to be upregulated in E21.5 CDH mvECs (**d**). Increased inflammation was not predicted by IPA for the NC mvECs (**e**). Rather, the NC mvECs were enriched for necrosis, apoptosis and notable for reduced cell viability. Activation states for the different biological processes were inferred from z-scores, which relate experimentally observed differential gene expression with literature-derived directions of effect to predict implicated biological functions independent of the associated p-values. Activation z-scores ≥|2| were considered most significant by convention.

Ingenuity Pathway Analysis (IPA) was used to analyze the DGE for CDH versus 2HC mvECs and for NC versus 2HC mvECs. Heatmaps of differentially expressed genes that were paired to relevant pathways in IPA are shown in Fig. 3a–c. Data are presented separately for genes that were differentially expressed in both the CDH and NC groups (Fig. 3a), only in the CDH group (Fig. 3b), and only in the NC group (Fig. 3c). In the CDH versus 2HC comparison, these genes predicted increased inflammation in CDH, including through pathways related to phagocytic cells, neutrophils and leukocytes that all exceeded an activation z-score of |2| (Fig. 3d). There were also significant signals for increases in production of reactive oxygen species (ROS) with activation z-scores not far below |2|.

The biological processes defined by the NC versus 2HC DGE were different. In Fig. 3e, both the most significant pathways and any pathways that could be matched to those in Fig. 3d were reported to provide the best contrast between the groups. Overall, the NC versus 2HC DGE was characterized by increased necrosis, apoptosis and cell death and reduced cell viability. Activation z-scores for biological processes related to inflammation were well below thresholds conventionally considered significant.

**Figure 4:**
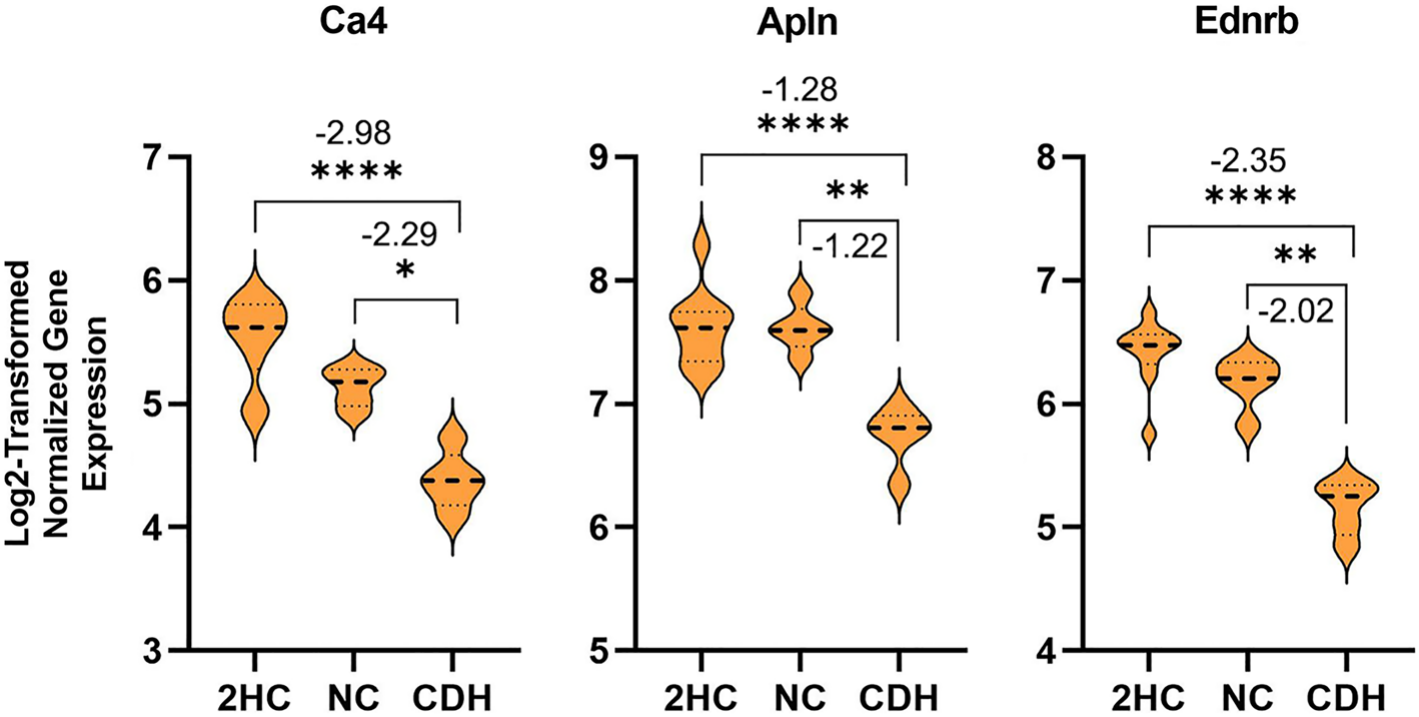
Lung compression led to downregulation of three genes within E21.5 mvECs that defined a subpopulation of endothelial cells relevant to alveolar formation and response to injury. CDH mvEC were compared to NC mvEC to detect differences that were most clearly related to lung compression instead of nitrofen-exposure. This comparison revealed reductions in carbonic anhdyrase IV (*Ca4*), apelin (*Apln*) and endothelin receptor type B (*Ednrb*) genes in CDH, which characterize a previously identified subpopulation of enodthelial cells that participate in gas exchange, alveolar formation and repair of lung injury. Asterixes identify statistically significant changes with the number of asterixes identifying how many significant zeros there are in the *p*-value. The number above or below the asterixes is the log_2_ fold change in the CDH cells.

Differentially expressed genes from the CDH versus NC comparison included carbonic anhydrase IV (*Ca4*, log2FC = − 2.29), endothelin receptor type B (*Ednrb*, log2FC = − 2.02), protocadherin alpha 13 (*Pcdha13*, log2FC = − 1.78) and apelin (*Apln*, log2FC = − 1.22) (Fig. 4). Differential expression of these genes was detected at our threshold when comparing CDH versus 2HC and CDH versus NC but not when comparing NC versus 2HC. While these genes are too few to analyze in IPA, three of these genes (*Ca4*, *Apln*, and *Ednrb*) are notable in that they are the key markers defining a previously described subpopulation of microvascular endothelial cells that participate in gas exchange, alveolar formation and repair of lung injury. As these genes were detected in our most direct and stringent analysis of the impact of lung compression and because they clearly defined a known subpopulation of endothelial cells, we chose to focus on *Ca4*^+^ cells.

### Examination of Ca4^+^ pulmonary endothelial cells

**Figure 5:**
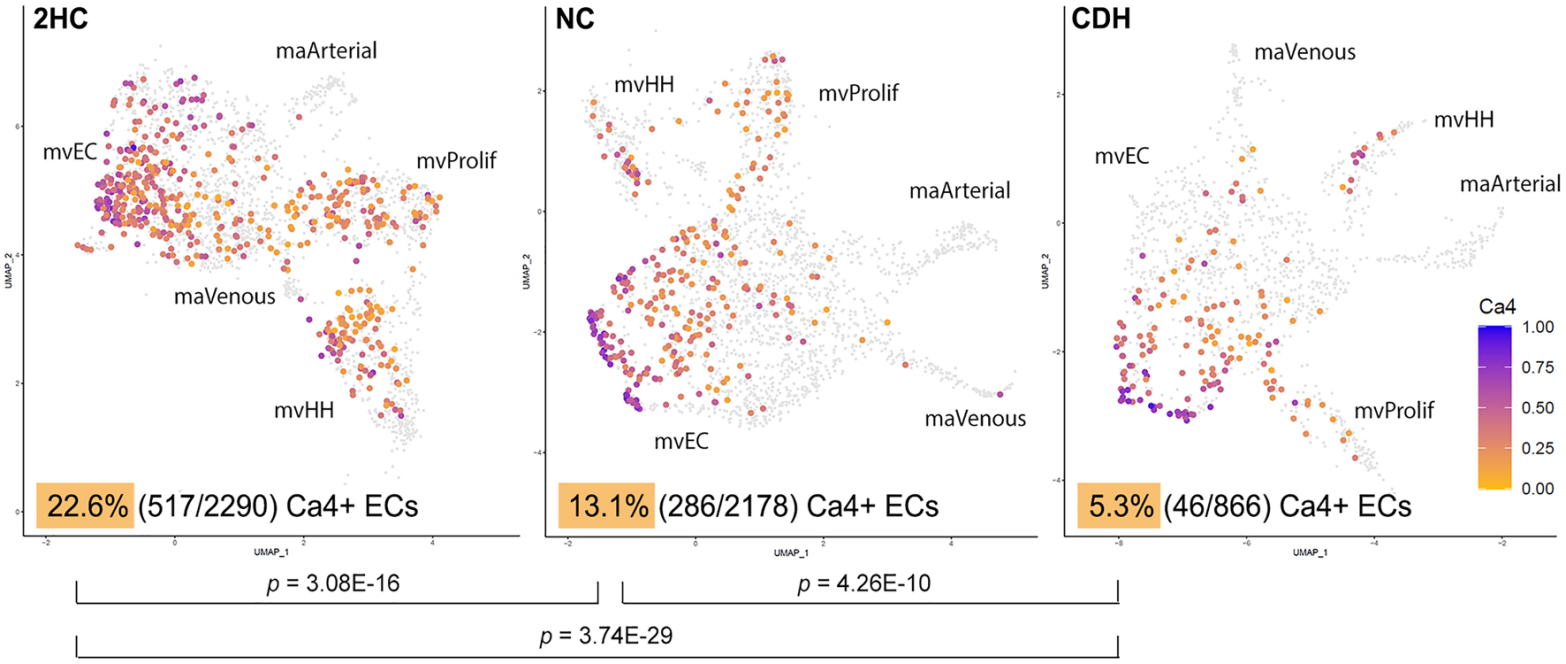
*Ca4*^+^ microvascular endothelial cells were significantly reduced in CDH. Carbonic anhydrase IV gene expression is shown as scaled UMAPs within the endothelial cell subclusters for the three experimental groups. *Ca4*^+^ cells were only detected within the microvascular subclusters as opposed to the two macrovascular clusters. Moreover, the percentage of cells with any *Ca4* expression was significantly reduced with nitrofen exposure and then again with lung compression, such that nitrofen exposure and lung compression in CDH have additive or synergistic effects in depleting the number of Ca4+ endothelial cells. Reported *p*-values are Bonferroni corrected proportion analyses.

To further examine *Ca4*^+^ endothelial cells, we sought to quantify these cells and identify their distribution in our three experimental groups. Scaled UMAPs showing *Ca4* gene expression within the endothelial cell clusters were generated (Fig. 5). As expected, *Ca4* expression was unique to the microvascular endothelial cell clusters (i.e. mvEC, mvProlif and mvHH). The majority of *Ca4*^+^ cells were located within the mvEC cluster, but many were also located within the mvProlif and mvHH clusters. Strikingly, the total number of *Ca4*^+^ endothelial cells was reduced from 22.6% in the 2HC clusters to 13.1% in the NC clusters (*p* = 3.08E−16). These cells were further reduced between the NC group and the CDH group (13.1 to 5.3%, *p* = 4.26E−10) (Fig. 5). It is also notable that the percentage of *Ca4*^+^ mvProlif cells dropped from 2HC to the two experimental groups. *Ca4*^+^ mvProlif cells account for 4.8% of total 2HC microvascular endothelial cells (110/2290), but they only account for 1.8% (39/2178) and 1.6% (14/866) of total NC and CDH microvascular endothelial cells, respectively (p < 0.0001).

The presence of *Ca4*^+^ microvascular endothelial cells (mvCa4+) have previously been shown to be dependent upon vascular endothelial growth factor (VEGF)-A expression specifically from type I alveolar cells (AT1). Therefore, we compared *Vegfa* expression between 2HC, NC and CDH type I alveolar cells, and no differences were observed (Supplemental Fig S4).

### Analysis of differential gene expression in Ca4^+^ microvascular endothelial cells

To determine if gene expression differences may account for the reduced number of mvCa4+ ECs, we examined DGE for these manually selected cells. Within our DGE cutoff of log_2_ fold change (log_2_FC) >|1|, there were 12 genes up- and 69 genes down-regulated in CDH versus 2HC mvCa4+ ECs, 11 genes up- and 32 genes down-regulated in NC versus 2HC mvCa4+ ECs, and no differential gene expression in CDH versus NC mvCa4+ ECs (Supplemental Excel File).

**Figure 6:**
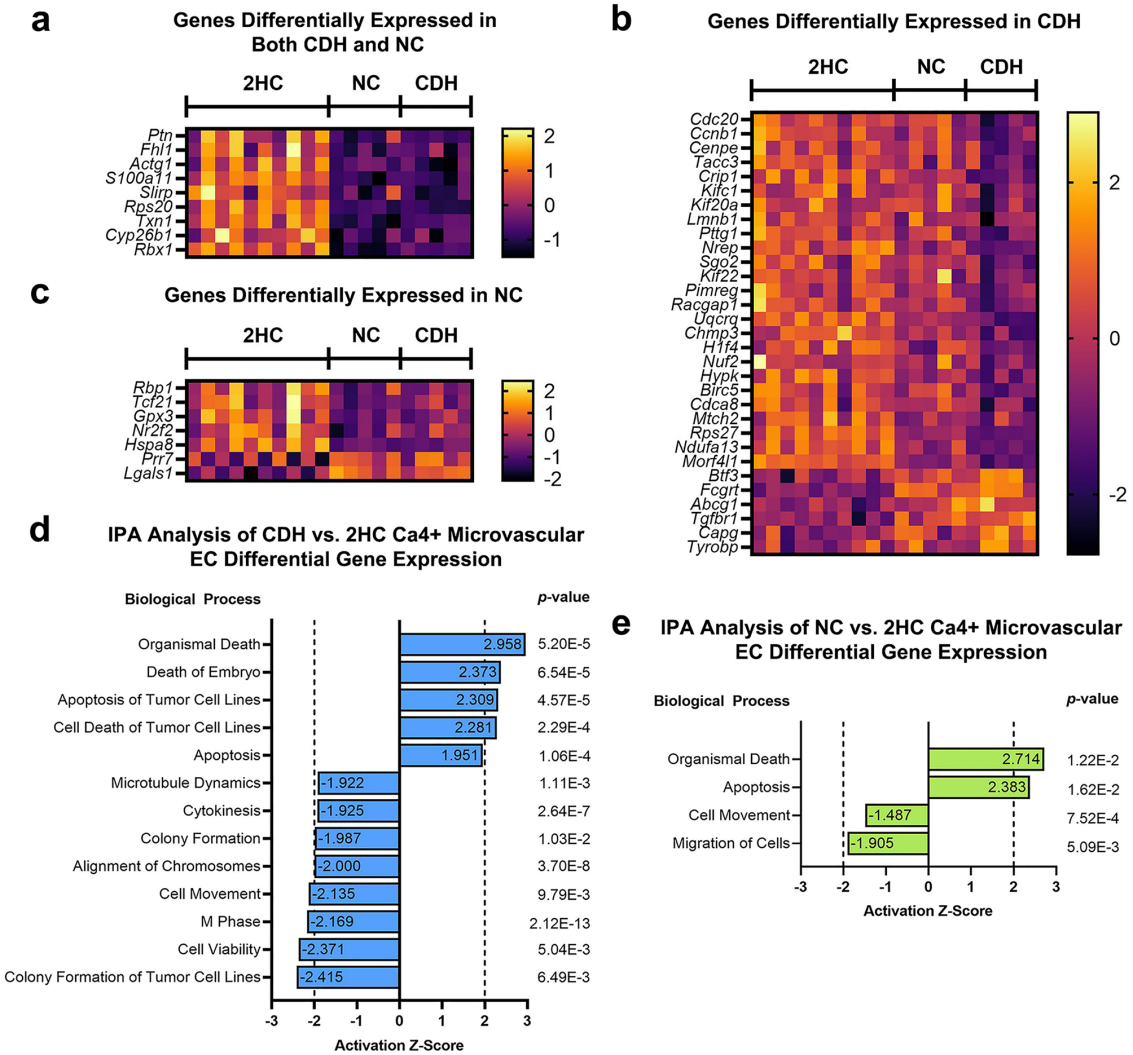
Differentially expressed genes within *Ca4*^+^ microvascular endothelial cells predicted reduced cell division, reduced cell movement and increased apoptosis in CDH. Differential gene expression was determined for both CDH and NC lungs compared to healthy controls and analyzed by Ingenuity Pathway Analysis (IPA). Expression of genes attached to biological pathways detected by IPA are shown in heatmaps as differentially expressed in both CDH and NC lungs (**a**), differentially expressed in only CDH lungs (**b**) and differentially expressed in only NC lungs (**c**). Differentially expressed *Ca4*^+^ microvascular endothelial cell genes identified in the heatmaps are sorted by smallest to largest log2FC. Several biological processes related to cell cycle and cell division were predicted by IPA to be uniquely downregulated in E21.5 CDH *Ca4*^+^ enodthelial cells (**d**). IPA predicted increased apoptosis, organismal death and cell movement in these cells from both the CDH and NC groups (**d, e**). Activation states for the different biological processes were inferred from z-scores, which relate experimentally observed differential gene expression with literature-derived directions of effect to predict implicated biological functions independent of the associated p-values. Activation z-scores ≥|2| were considered most significant by convention.

IPA was used to further analyze the DGE for CDH versus 2HC mvCa4+ ECs and for NC versus 2HC mvCa4+ ECs. Expression of differentially expressed genes that were paired to relevant pathways in IPA are reported in Fig. 6a–c. Data are presented separately for genes that were differentially expressed in both the CDH and NC groups (Fig. 6a), only in the CDH group (Fig. 6b), and only in the NC group (Fig. 6c). In the CDH versus 2HC comparison, these genes uniquely predicted decreased processes relevant for cell division, including cytokinesis (activation z-score = − 1.925), alignment of chromosomes (− 2.000), and M phase (− 2.169) (Fig. 6d). Both the CDH versus 2HC and NC versus 2HC IPA analyses suggested increases in apoptosis and organismal death and decreases in cell movement, but no biological pathways relevant to cell division were detected in the NC versus 2HC analysis (Fig. 6d, e).

### Endothelial cell subpopulation gene ontology

**Figure 7:**
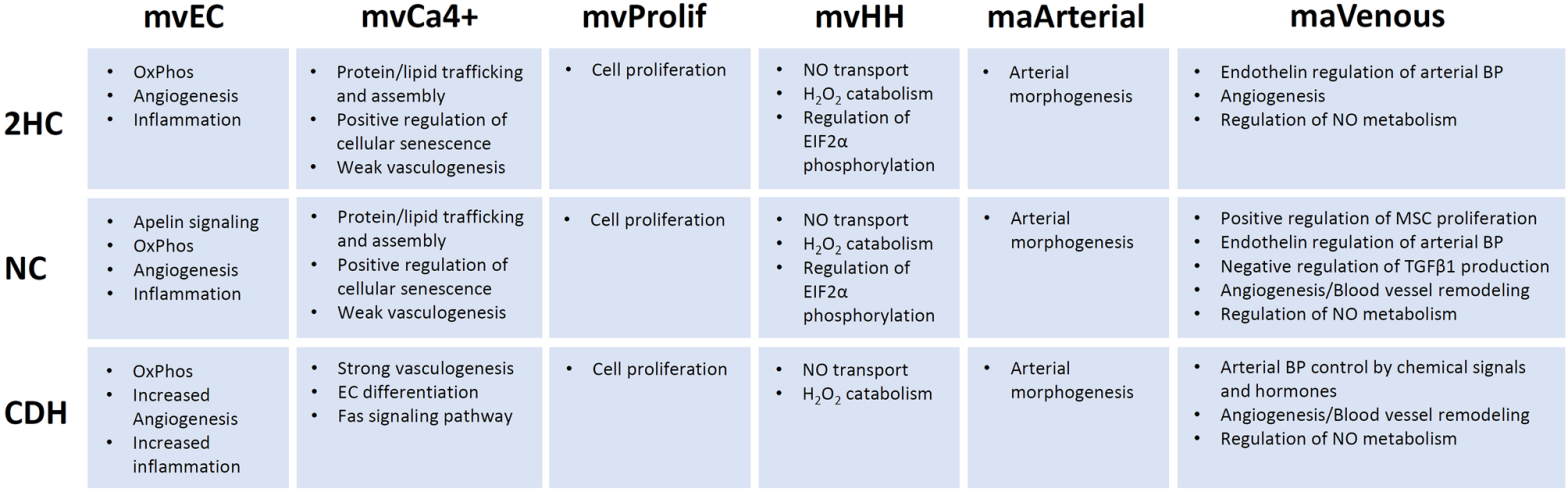
Gene ontology analysis predicted increased vasculogenesis in microvascular Ca4^+^ endothelial cells and increased inflammation in mvECs. Endothelial cell clusters were compared to each other within each experimental group using PANTHER gene ontology analysis. Results are summarized above. BP = blood pressure, EC = endothelial cell, MSC = mesenchymal stem cells, OxPhos = oxidative phosphorylation, NO = nitric oxide.

Gene ontology (GO) was examined for each EC subpopulation to provide further insight into their biological functions and to determine if biological functions differed between experimental groups. These data are presented in detail for mvCa4+ cells in Supplemental Figure S5 and for mvECs in Supplemental Tables S1, S2 and S3, and they are summarized for all endothelial cell subclusters in Fig. 7. GO analysis of the mvEC population was remarkable for enrichment of angiogenesis in all three groups but with a significant increase in the CDH group. Inflammation was also significantly increased in the mvEC CDH group, largely manifest by endogenous antigen presentation. Notably, the mvEC NC group was enriched for apelin signaling, which is notable since *Apln* was one of the downregulated genes between NC and CDH mvECs.

Notably, 2HC and NC mvCa4+ ECs were primarily enriched for protein and lipid trafficking and assembly, such as plasma membrane raft assembly, SNARE complex assembly and vesicle fusion with Golgi apparatus (Supplemental Fig. S5). There were relatively weak signals for regulation of vasculogenesis that did increase somewhat from 2HC to NC (fold enrichment = 5.68 vs. 16.42, respectively). However, regulation of vasculogenesis had a 28.25 fold enrichment in CDH cells, along with strong enrichment of several other biological process pathways related to blood vessel endothelial cell differentiation, cell migration involved in sprouting angiogenesis, basement membrane organization, and vascular endothelial growth factor signaling pathways.

The GO signature for the mvProlif and maArterial endothelial cells did not differ between experimental groups. GO for the mvHH group suggested that this novel cluster may have roles in nitric oxide transport or hydrogen peroxide catabolism. Finally, the maVenous gene ontology suggested that these cells play important roles in regulation of nitric oxide metabolism, regulation of arterial blood pressure and angiogenesis. Some unique pathways related to positive regulation of mesenchymal stem cell proliferation were noted in the NC maVenous group. However, the greatest differences were among in the mvEC and mvCa4+ cell groups.

## Discussion

This study revealed heterogeneity of saccular stage microvascular endothelial cells and demonstrated that lung compression results in depletion of *Ca4*^+^ microvascular endothelial cells in the nitrofen rat model of CDH. Gene ontology suggested that *Ca4*^+^ cells were primed for vasculogenesis with lung injury in CDH, but their differential gene expression patterns suggested downregulation of processes related to cell division. That may indicate that limitations in proliferation result in their relative depletion in CDH, thereby impairing their intended function. In contrast, a population of general microvascular endothelial cells low in *Ca4* expression has a transcriptomic profile suggesting that it mediates the inflammatory response. Additional populations include highly proliferative microvascular endothelial cells and a novel cluster high in hemoglobin. Since endothelial cell heterogeneity has never been explored in CDH, this work provides a better understanding of microvascular endothelial cell subtypes that are active in pathogenesis and could be leveraged to develop novel therapeutics targeting specific components of the endothelium^36^.

Recent work in murine lungs has demonstrated capillary cell-type specialization in the alveolus, including a population of microvascular endothelial cells characterized by plasma membrane carbonic anhydrase IV^15^. These cells develop gradually and asynchronously with other microvascular endothelial cell subtypes after first emerging during the saccular stage of fetal lung development^15^ and are dependent on *Vegfa* expression from type I alveolar cells^18^. They have been called “aerocytes” based on their expression of genes related to gas exchange and the fact that they are separated from type I alveolar cells by only a limited basement membrane and without intervening pericytes. However, that nomenclature may not reflect the full scope of their role^15,18,27^. *Ca4*+ microvascular endothelial cells have been shown to preferentially localize to regenerating areas of the alveolus after lung injury, suggesting a prominent role in alveolar repair^27^. Moreover, without these cells, the alveolar space is aberrantly enlarged^18^. In our study, we show that lung compression uniquely downregulates genes that characterize these cells and leads depletion of *Ca4*^+^ microvascular ECs within the developing lung. This is the first study that has looked at endothelial heterogeneity in CDH and the first that has shown this change. The consequences of loss of these cells require further study, but it is reasonable to hypothesize that mvCa4+ EC deficiency affects gas exchange, alveolar development, and/or response to alveolar injury in CDH. How these findings translate to human disease remains an open question. However, this finding is further notable as it is one of the few differences that was strong enough to emerge from our direct comparison between CDH and NC mice. Such findings that are more clearly related to lung compression rather than nitrofen are more likely to be translatable to human disease. *Ca4* is present in pulmonary endothelium in other mammals, including humans where the enzyme is present during lung development and more active^15,17^, raising the possibility of conserved functions similar to those that have been identified in murine models.

Apelin and endothelin receptor type B are also noteworthy as their expression is part of the transcriptomic signature of mvCa4+ ECs. Mice deficient in apelin demonstrate significant pruning of the microvasculature and worse pulmonary hypertension with hypoxia^37^, and apelin expression is decreased in plasma and pulmonary endothelial cells in humans with pulmonary hypertension^38^. This is in part because apelin modulates endothelial nitric oxide synthase (eNOS) expression^37^, induces eNOS-dependent vasodilation, counteracts angiotensin II mediated vasoconstriction and has positive inotropic and cardioprotective effects^38^. These findings are consistent with the observed phenotype of our model, and previous work has confirmed our findings by showing via real-time PCR and western blotting that apelin and apelin receptor are decreased in nitrofen induced CDH at E21.5^39^. However, our work expands upon the prior work in the nitrofen rat by showing that lung compression rather than nitrofen leads to downregulation of apelin specifically in the microvasculature. Our gene ontology analysis also shows that nitrofen-exposed microvascular endothelial cells are enriched in apelin signaling in the absence of lung compression from an ipsilateral diaphragmatic hernia. It is worth noting that our data show that apelin is more widely expressed in the endothelium than just within the mvCa4+ ECs, particularly within the mvProlif ECs, but it is possible that reductions in *Ca4*^+^/*Apln*^+^ ECs contribute to the many effects of apelin in disease.

The role of endothelin receptor type B in CDH microvasculature is less clear. Previous work in the nitrofen rat has shown that endothelin receptor type B is increased in CDH pulmonary arteries from E15–E21; however, those studies did not examine the microvasculature where we have observed differences. Our data do not show a difference in transcription of *Ednrb* in maArterial cells, but we do demonstrate that *Ednrb* is highly expressed in the macrovasculature, even more so in maVenous cells than maArterial cells, and so our data do not exclude the possibility, that there could be differences between the micro- and macro-vasculature. Different expression patterns between micro- and macrovasculature are consistent with what has been shown for other genes^14^ and provides even more reason to understand the varied nature of the functional and anatomic heterogeneity in the pulmonary endothelium. Differential expression of *Ednrb* between the macro- and microvasculature could explain why treatment with endothelin receptor antagonists significantly reduces pulmonary artery pressure while pulmonary artery flow and pulmonary vascular resistant remain constant in surgical models of CDH (which rely solely on the impact of lung compression)^40^. *Ednrb* activity has also been shown to influence angiogenesis in multiple disease systems, and such mechanisms could be at play in the microvasculature in CDH apart from the vasoconstrictive effects in the microvasculature^41–44^.

Furthermore, our work suggests that the general population of microvascular endothelial cells that are low in *Ca4* (mvECs) are the primary mediators of inflammation and generation of reactive oxygen species in the microvasculature. It is well known that CDH is, in part, an inflammatory disease with further damage inflicted by oxidative stress^45–49^. However, the idea that specific subsets of endothelial cells could mediate these changes is relatively new with the advent of single cell transcriptomics. Microvascular endothelial cells lacking *Ca4* encode genes for MHC class II components, suggesting that they play a role in antigen presentation. Moreover, in chemically induced rat pulmonary inflammation, *Ca4*-high endothelial cells are mainly responsible for angiogenesis, while *Ca4*-low endothelial cells are involved in neutrophil recruitment^16^. This is consistent with our IPA analysis that revealed transcriptomic signatures for biological processes involving infiltration by neutrophils and recruitment of phagocytic cells in CDH mvECs. While these differences were not detected in the CDH versus NC DGE, they were not present in nitrofen exposed controls, suggesting that they were influenced by lung compression. The fact that there is an apparent “division of labor" within heterogenous populations of microvascular endothelial cells suggests that certain endothelial processes could be specifically targeted by leveraging endothelial subclusters.

Another microvascular EC group we detected was the novel mvHH subcluster that was considerably depleted in nitrofen exposed lungs in both the NC and CDH group. The trend towards further losses of these cells between the NC and CDH groups just narrowly missed statistical significance. The function of this cluster is uncertain, but it is interesting to speculate based off gene ontology analysis that it may play a role in nitric oxide regulation or hydrogen peroxide catabolism. Hemoglobin subunit alpha (Hba) is expressed by ECs and enriched in the myoendothelial junction (MEJ), where it regulates vascular tone and function through control of nitric oxide (NO) levels by reduced heme iron^50^. Neither the presence of these cells nor their loss in disease have been previously described. Finally, proportions of proliferative microvascular endothelial cells are also reduced by nitrofen exposure, but we have not detected any noteworthy differences in these cells between the NC and CDH groups.

There are limitations to our study. First, our approach, which required fresh single cell preparations, did not allow us to histologically phenotype lungs ipsilateral to the diaphragmatic defects prior to sequencing. This may have led to some variability in the selected lungs; however, our assumption was that selecting the smallest lungs by gross phenotyping also selected the most severely diseased lungs. Second, our approach of sequencing the whole lung, rather than limiting our analysis to only endothelial cells meant that our sample size was smaller for rare clusters. The positive aspects of that approach are that we did not risk losing more cells during a longer preparation or isolation step, which may also have resulted in loss of rare clusters, and that it allowed us to examine interactions between endothelial cells and non-endothelial cell types. Third, because Vireo works with genotypes (and in this case from a largely genetically homogenous set of donors) and is probabilistic in nature, it is a less robust approach than having individual replicates or using cell multiplexing. We did not detect more differences in the CDH versus NC DGE comparisons, in part, due to closeness of the means and/or high variance in one or both groups. This may have been different with more robust replicates. Fourth, our data only represent “potential” of what the cells are capable of based on the transcriptome. We do not present functional or protein expression data in this study. Our data also cannot demonstrate a causative link between mvCa4^+^ endothelial cell reduction and lung phenotype. Nevertheless, it is the first report of scRNA-seq applied to the study of CDH in any model, and, as such, our data is unbiased and represents the most comprehensive report of endothelial cell heterogeneity in CDH.

In conclusion, we have identified 3 unique subclusters of microvascular endothelial cells for which gene ontology analysis predicts unique functions. Our results demonstrate that lung compression from CDH results in loss of *Ca4*^+^ microvascular endothelial cells, which have been previously shown to have an important role in normal alveolar development, gas exchange and response to injury. Notably, the primary drivers of inflammation in the endothelium appear to be the *Ca4*-low mvECs. These data reveal endothelial cell heterogeneity during late-stage fetal development in CDH and begin to provide a roadmap for developing therapeutics that target the endothelium^36^. These abnormalities may be foundational to both pulmonary hypoplasia and pulmonary hypertension in CDH, and the saccular stage of development is one in which prenatal treatment could be most readily administered.

## Methods

### Nitrofen model

Cleveland Clinic Institutional Animal Care and Use Committee (IACUC) approval was obtained (Protocol #2019–2282), and experiments were performed in accordance with all relevant guidelines and regulations for the care and use of laboratory animals. This study was carried out in compliance with the Animal Research: Reporting of In Vivo Experiments (ARRIVE) guidelines. Time-mated, pregnant Sprague–Dawley rats (Envigo, Hackensack, NJ) with a mean ± SD weight of 230.3 ± 5.2 g were gavage fed 100 mg nitrofen (2,4-diclorophenyl *p*-nitrophenyl-ether; Sigma Aldrich, St. Louis, MO) dissolved in olive oil on embryonic day 9.5 (E9.5) to induce fetal diaphragmatic hernia, pulmonary hypoplasia and pulmonary hypertension that is similar to human disease^51^. 1 mL olive oil alone was gavage fed to healthy controls. Pregnant dams were euthanized with CO_2_ asphyxiation and subsequent cervical dislocation. Fetuses were immediately harvested by cesarean section on E21.5 and decapitated prior to proceeding with dissection to examine the diaphragm and isolate the left lung. Only fetuses with left-sided diaphragmatic defects > 50% of the area of the diaphragm were selected for analysis in the CDH group. We achieved on average 73% of fetuses with left-sided diaphragmatic defects. Lungs from nitrofen exposed fetuses without a diaphragmatic defect were analyzed as a second control population in order to distinguish the effect of nitrofen from that of lung compression. Left lungs (those ipsilateral to diaphragmatic defect in the CDH group) from all three experimental groups were carefully dissected free of all surrounding structures (including the heart, aorta, esophagus, trachea, and right lung) in cold PBS using 2.5x loupe magnification.

### Lung phenotyping

Lungs were phenotyped by measuring weight, length and width. For control lungs, the largest lungs were selected for scRNA-seq. For the CDH group, we selected left lungs that were smallest in relation to the matched right lung in order to choose ones that were most impacted by left lung compression. There were fewer NC lungs to select from, but the largest ones were used for scRNA-seq. Ratios of left-to-right lung length were calculated from photographs.

Formalin-fixed left lungs were processed using a Leica Peloris 3 automated processor (Leica Biosystems, Deer Park, IL), embedded in paraffin, sectioned and stained with hematoxylin and eosin for histological analysis. The CDH and 2HC lungs were taken from littermates of fetuses selected for scRNA-seq, but NC lungs were from a subsequent litter due to the relative paucity of NC lungs. Images of tissue sections were acquired using an AT2 whole slide scanner (Leica Biosystems) at 20x magnification. Image analysis was performed within ImageScope version 12.4.3.5008 (Leica Biosystems) and ImageJ 1.53t, which is open source software.

### Single cell preparation and scRNA-sequencing

Pooled NC and CDH specimens combined fetuses from two separate pregnant dams, and the two sets of pooled 2HC specimens each came from a different mother. Pooled lungs were digested into a single cell suspension, as previously described^52^. Briefly, lung tissue was minced and dissociated in 0.1% collagenase A (Sigma Aldrich), 0.04% DNase I (Sigma Aldrich), and 0.5 mM CaCl_2_ in 3 ml of 10 mg/mL Dispase II (Sigma Aldrich). Incubations were performed on an orbital shaker at 37 °C for a total of 45 min. Cell clumps were disrupted via pipetting, and the suspension was passed through a 40-micron cell strainer to remove any undigested tissue pieces or aggregates. Pelleted cells were then resuspended in 1% BSA in PBS to prevent aggregation. Cell viability and concentration were assessed using propidium iodide and acridine orange with a CellDrop automated cell counter (DeNovix, Wilmington, DE).

A quantity of 10,000 single cells were targeted using the 10 × Genomics Chromium Controller (Pleasanton, CA) for cDNA synthesis and barcoding. cDNA quality and quantity of each sample were assessed using a Bioanalyzer High Sensitivity DNA assay, and samples were constructed following the 10 × Genomics Chromium Single Cell 3’ V3 protocol (CG000183 Rev A). Sample libraries were then assessed for proper construction using the same Bioanalyzer assay. Samples were pooled equally, and the resulting DNA library was quantified using the sparQ Universal Library Quantification kit (Quantabio, Beverly, Massachusetts) to inform sample preparation for downstream Illumina sequencing. Libraries were denatured and sequenced on an Illumina Novaseq 6000 (San Diego, CA) high-throughput sequencing platform with 28 cycles for the forward read and 91 cycles for the reverse read. Each cell was sequenced to a minimum of 20,000 reads per cell per manufacturer’s recommendations. We achieved an average sequencing saturation of 33.1%.

### Bioinformatics analysis

BCL files from the Novaseq were converted to FastQ files with the Cell Ranger (10 × Genomics Cell Ranger 4.0.0) mkfastq function^53^. An alignment reference was constructed using the Cell Ranger mkref function applied to the Ensembl *Rattus norvegicus* genome fasta (http://ftp.ensembl.org/pub/release-104/fasta/rattus_norvegicus/dna/Rattus_norvegicus.Rnor_6.0.dna.toplevel.fa.gz) and GTF (http://ftp.ensembl.org/pub/release-101/gtf/rattus_norvegicus/Rattus_norvegicus.Rnor_6.0.101.gtf.gz, filtered with the Cell Ranger mkgtf function with option “—attribute = gene_biotype:protein_coding”). Reads were aligned and genes quantified with the Cell Ranger count function.

Downstream analysis was performed with R (version 4.1.0.)^54^ and Seurat (version 4.0.3)^55^. Default options for each function were used unless otherwise specified. Each group of samples was analyzed separately. Samples were filtered to exclude cells with > 20% mitochondrial expression, < 500 genes per cell, < 500 unique molecular identifiers (UMI) per cell, and a complexity score (log_10_[genes per cell/UMI per cell]) < 0.8. All samples were filtered to exclude genes expressed in less than 10 cells. To detect and remove doublets from each sample, FreeBayes (version 1.3.4)^56^^2^ was used to call germline variants from the Cell Ranger BAM files, using the same genome reference as used for alignment. These variants were then filtered for cross-cell total allele counts of ≥ 100 using bcftools (version 1.12)^57^^3^ and cell doublet assignments were calculated with Variational Inference for Reconstructing Ensemble Origins (Vireo, version 0.5.6)^54^. UMI counts were normalized with Seurat function SCTransform^58^, using method “qpoisson”, and additionally corrected for percent mitochondrial expression.

The 2 healthy control samples were integrated with functions SelectIntegrationFeatures (using and returning the top 3000 most variable genes), PrepSCTIntegration, FindIntegrationAnchors (using normalization method “SCT” and 5 anchors), and IntegrateData (using normalization method “SCT”). Principal component analysis (PCA) was performed on the integrated counts with function RunPCA. Clusters were identified using functions FindNeighbors (using the first 30 PCs) and FindClusters (using a range of resolutions from 0 to 1.0 at every 0.05 increment). Marker genes conserved across the 2 healthy control samples were identified for each cluster with function FindConservedMarkers and annotated with R package AnnotationHub (version 1.54.1)^59^. Marker gene lists were manually analyzed to determine the cell type of each cluster. For the nitrofen control and nitrofen CDH samples, PCA was performed on the SCT-normalized counts; clustering and marker analysis was performed for each sample separately, using a resolution of 1.0 with FindClusters. Uniform Manifold Approximation and Projection (UMAP) was applied to the first 30 principal components (PCs) with function RunUMAP. Of note, manually selected *Ca4*^+^ endothelial cells were selected based on any *Ca4* expression above zero.

### Composition analysis

Differences in cell proportion of a given cell type between two experimental groups (integrated healthy controls, nitrofen control, nitrofen CDH) was performed with function prop.test in base R, using the total cell counts of each group as the number of trials and the cell type count as the number of successes. Two-sided tests were performed and Bonferroni correction was applied to adjust for multiple testing using function p.adjust in base R.

### Donor analysis

To enable reconstruction of sample identities for each cell in the pooled specimens of fetal lungs, genetic variants that segregate between the samples were used as natural barcodes for cell demultiplexing. Germline polymorphic variants were inferred from scRNA reads in each cell and aggregated across cells, as described above. In doing so, these incomplete genotype data were sufficient to reconstruct a partial genotypic state for rats in the pooled specimens. The filtered genotypes, along with the known number of pooled fetal rat lungs for each sample, were analyzed with Vireo^60^. Four pooled specimens were used, as follows: CDH = 5 lungs, NC = 5 lung, healthy control 1 = 5 lungs, and healthy control 2 = 5 lungs. Together, the two healthy controls were the 2HC group. Vireo estimated per-cell donor assignment probabilities based on distinct germline genotype patterns that were representative of unique lung samples, allowing each cell to be assigned to an arbitrarily named “donor” after probabilistic demultiplexing^60^. Donor cell assignment probabilities are summarized in Supplemental Figure S6. Cells for which there were equal per-donor probabilities were randomly assigned to donors.

### Experimental group differential gene expression

Our analytic approach to differential gene expression was designed to allow us to best distinguish the effect of nitrofen exposure from the effect of lung compression without statistical averaging that would not make sense biologically. Gene expression differences between experimental groups were evaluated for each cell type with the following permutations: CDH versus 2HC, NC versus 2HC and CDH versus NC. Pseudo-bulk counts (summed gene counts within each cell type) were generated for each donor within each cell type, using function aggregate.Matrix from R package Matrix.utils (version 0.9.8.)^61^. Differential gene expression analysis was performed with R packages edgeR (version 3.34.0)^62^ and limma (version 3.48.1)^63^, using default options, unless otherwise indicated. Pseudo-bulk counts were filtered for low expression with function filterByExpr, normalized for sequencing depth (UMI counts) and composition with function calcNormFactors (using method “trimmed mean of M-values”), and precision weights were estimated with function voom. Per-gene linear regression models were fit with a covariate for experimental group using function lmFit and contrasts for each comparison were calculated with functions makeContrasts and contrasts.fit. T-statistics from the regression models were moderated across genes with function eBayes. Multiple gene testing correction was applied using the Benjamini–Hochberg method and *p*-values were obtained from function topTable. An adjusted *p* < 0.05 was considered significant.

### Visualization

Pseudo-bulk counts were transformed for visualization with function regularized logarithm transformation (rlog) from R package DESeq2 (version 1.32.0)^64^. This was blinded to the experimental group. The rlog transformation functions similarly to a log_2_ transformation for genes with high counts but shrinks together values for different samples for genes with low counts, thereby stabilizing the variance between genes and rendering the data homoskedastic. Violin plots were generated from these rlog-transformed counts using Prism (version 9.1.2, GraphPad, San Diego, CA). Rlog-counts were additionally scaled with function scale from the base R package, so that each gene was adjusted by subtracting its mean and dividing by its standard deviation. The scaled rlog counts were used to produce heatmaps with Prism. To generate dot plots of the top marker genes for a given cell type, the percentage of cells expressing a gene and its z-score were calculated. Raw gene counts were normalized for UMI counts per cell and log-transformed with function NormalizeData from Seurat. Within each cell type, the exponentials (using function expm1 from base R) of the log-transformed values were used to generate an average and standard deviation, and then the normalized counts were scaled by subtracting the average and dividing by the standard deviation to create a z-score for each gene. Dot plots were generated using JMP Pro 16.2.0 (SAS, Cary, NC).

### Ingenuity pathway analysis

Expression analysis based on the expression log ratio was performed within Ingenuity Pathway Analysis (version 90348151, Qiagen, Redwood, CA). Queries looked at direct and indirect relationships of experimentally observed data across human, mouse and rat species and all available cell types. Expression log ratio cutoffs were set at a log_2_FC >|1| and an adjusted expression *p*-value of < 0.05. A disease or functional pathway was predicted to have directionality (up- or down-regulation) to its activation state if the activation z-score was ≥|2| per IPA standards^65^. Our analysis focused on biological processes relevant to angiogenesis, vessel formation, inflammation, cell division/proliferation, and endothelial cell function.

### Gene ontology analysis of endothelial cell subclusters

Marker genes for endothelial cell sub-clusters were investigated for Gene Ontology classification of biological processes with PANTHER (version 17.0)^66^. Results were adjusted for False Discovery Rate (FDR) as calculated by the Benjamini–Hochberg procedure, filtered for an overall FDR < 0.05, and sorted hierarchically to help understand the relationships between over-represented or enriched functional classes. *P*-values representing the probability that the number of genes observed in each category occurred by chance were determined by Fishers exact test or Binomial statistic, with a cutoff for significance of *p* < 0.05. These were represented graphically as − log(*p* value). The Fold Enrichment for each biological process of calculated by dividing the genes observed in the marker gene list over the expected number of genes for each category. Fold enrichment > 1 indicated overrepresentation in the data. Gene ratios were calculated by dividing the number of genes represented in our data from each category over the number of genes in the reference list that map to the particular annotation data category. Biological process enrichment dot plots were created using JMP Pro 16.1.0.

### Sample size analysis

The Genomics Core targeted 200 million reads per sample, which equated to an estimated 40 million expected reads per donor if shared amongst 5 donor lungs. Assuming endothelial cells would comprise a proportion ranging from 1 to 25% of total cells and that the expected reads would be divided across 25,000 possible genes, we anticipated on average a range of 16 to 400 per-gene pseudobulk counts. Assuming a coefficient of variation of 0.1 for rat samples, at 80% power and a per-gene alpha of 0.05/25,000, for equally sized groups (n = 5 donors), we expected to be able to detect a minimum fold change in mean endothelial cell gene expression ranging from 1.49 to 2.6. These assumptions were made with the knowledge that actual UMI counts would be less after accounting for PCR duplicates and that the number of expressed genes would be fewer based on 10x capture bias. Calculations were performed with function “rnapower” from R package RNASeqPower (version 1.34.0)^67^.

## Supplementary Information

Supplementary Information 1.

Supplementary Information 2.

## Data Availability

The datasets generated and analyzed during the current study are available in the NCBI Gene Expression Omnibus (GEO) data repository (https://www.ncbi.nlm.nih.gov/geo/, accession number GSE196313, currently available via token).
