# Supplementary material for: Single-cell transcriptomic profiling of microvascular endothelial cell heterogeneity in congenital diaphragmatic hernia
Source: Sci Rep. 2023 Jun 17;13:9851. doi: 10.1038/s41598-023-37050-y (PMC10276841; doi:10.1038/s41598-023-37050-y)
Supplement: Supplementary file 2 — Supplementary Information 2. [file 41598_2023_37050_MOESM2_ESM.pdf]

Single-cell transcriptomic profiling of microvascular endothelial cell heterogeneity in congenital diaphragmatic hernia

Jason O. Robertson, M.D., M.S. [1*], Peter Bazeley, M.D., M.S. [2], Serpil C. Erzurum, M.D. [3], Kewal Asosingh, Ph.D. [3]

[1] Cleveland Clinic Children's, Department of Pediatric Surgery, Cleveland, 44195, USA

[2] Cleveland Clinic Lerner Research Institute, Department of Quantitative Health Sciences, Cleveland, 44195, USA

[3] Cleveland Clinic Lerner Research Institute, Department of Inflammation and Immunity, Cleveland, 44195, USA

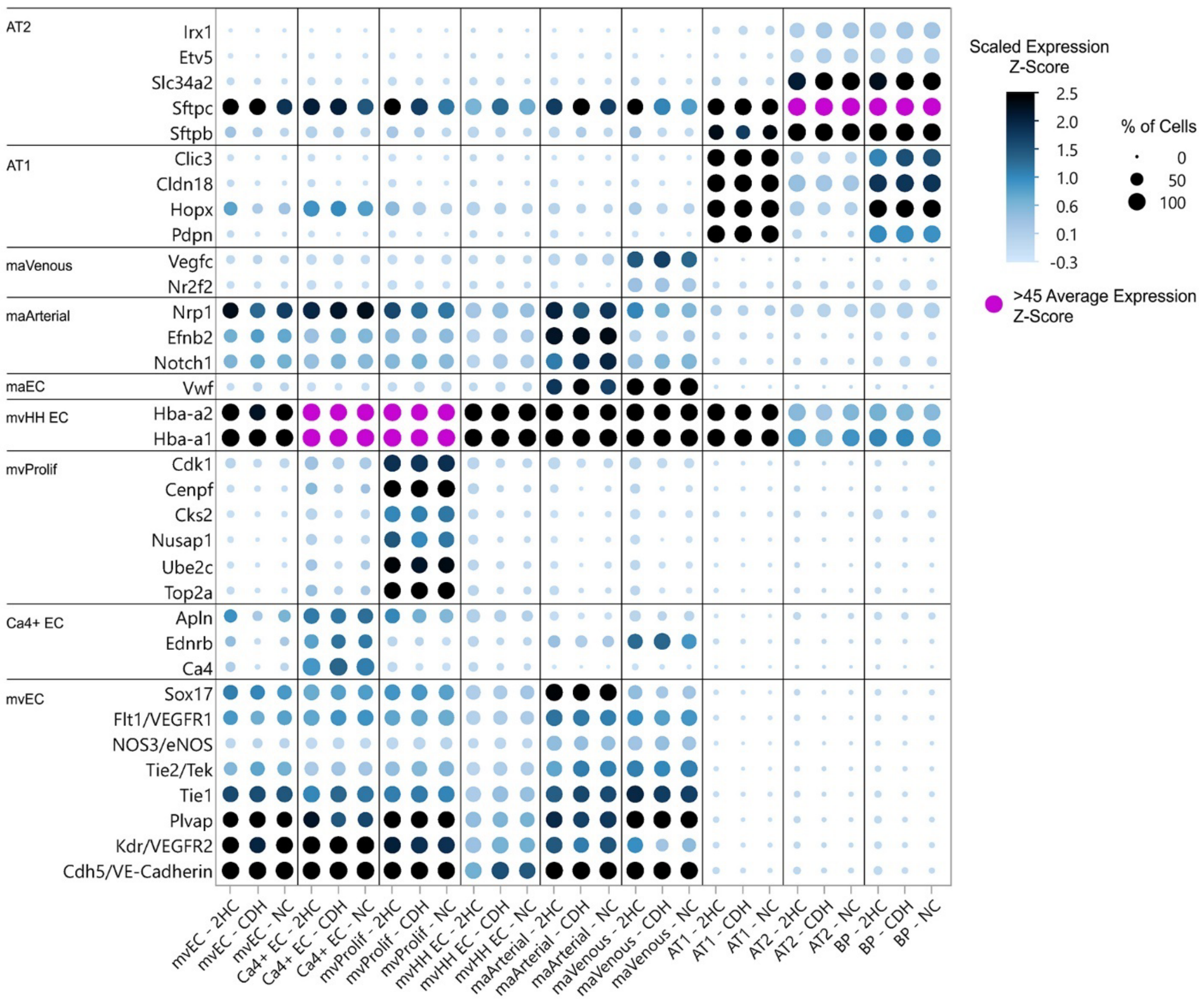

Supplemental Figure S1. Known cell type markers identified discrete endothelial and epithelial cell clusters within each experimental group.

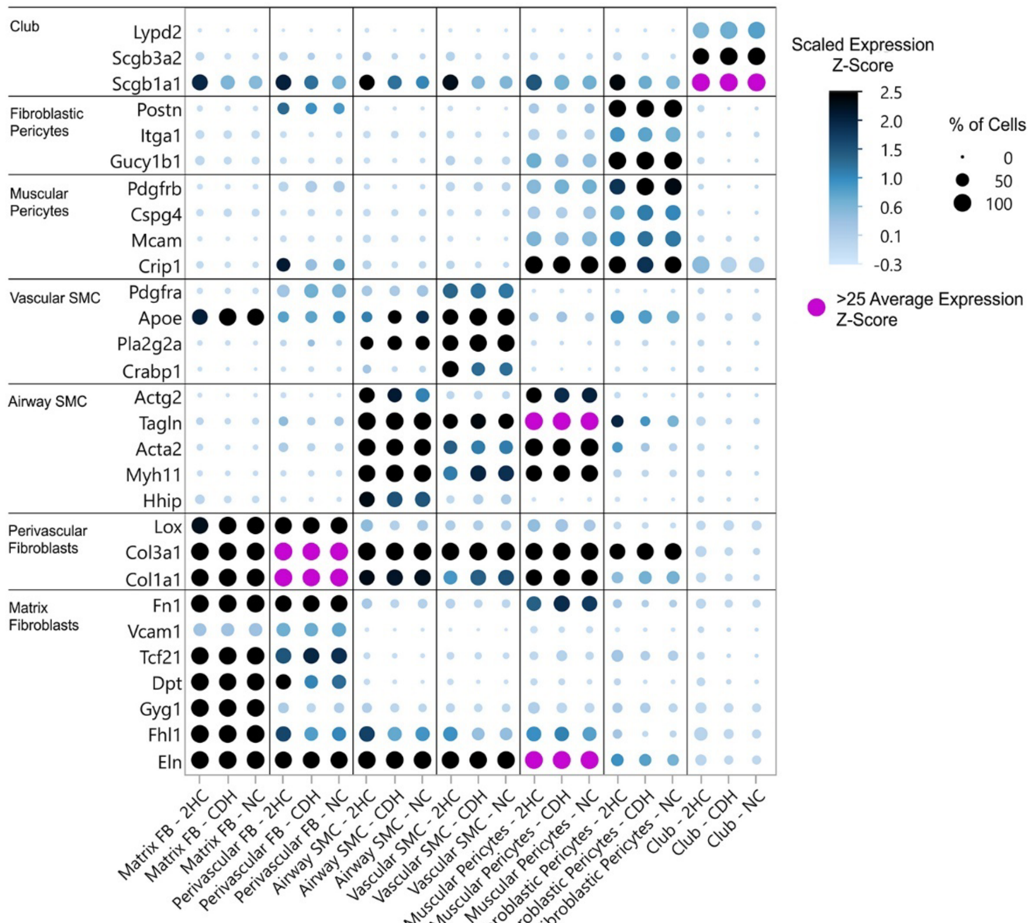

Supplemental Figure S2. Known cell type markers identified discrete mesenchymal and other cell clusters within each experimental group.

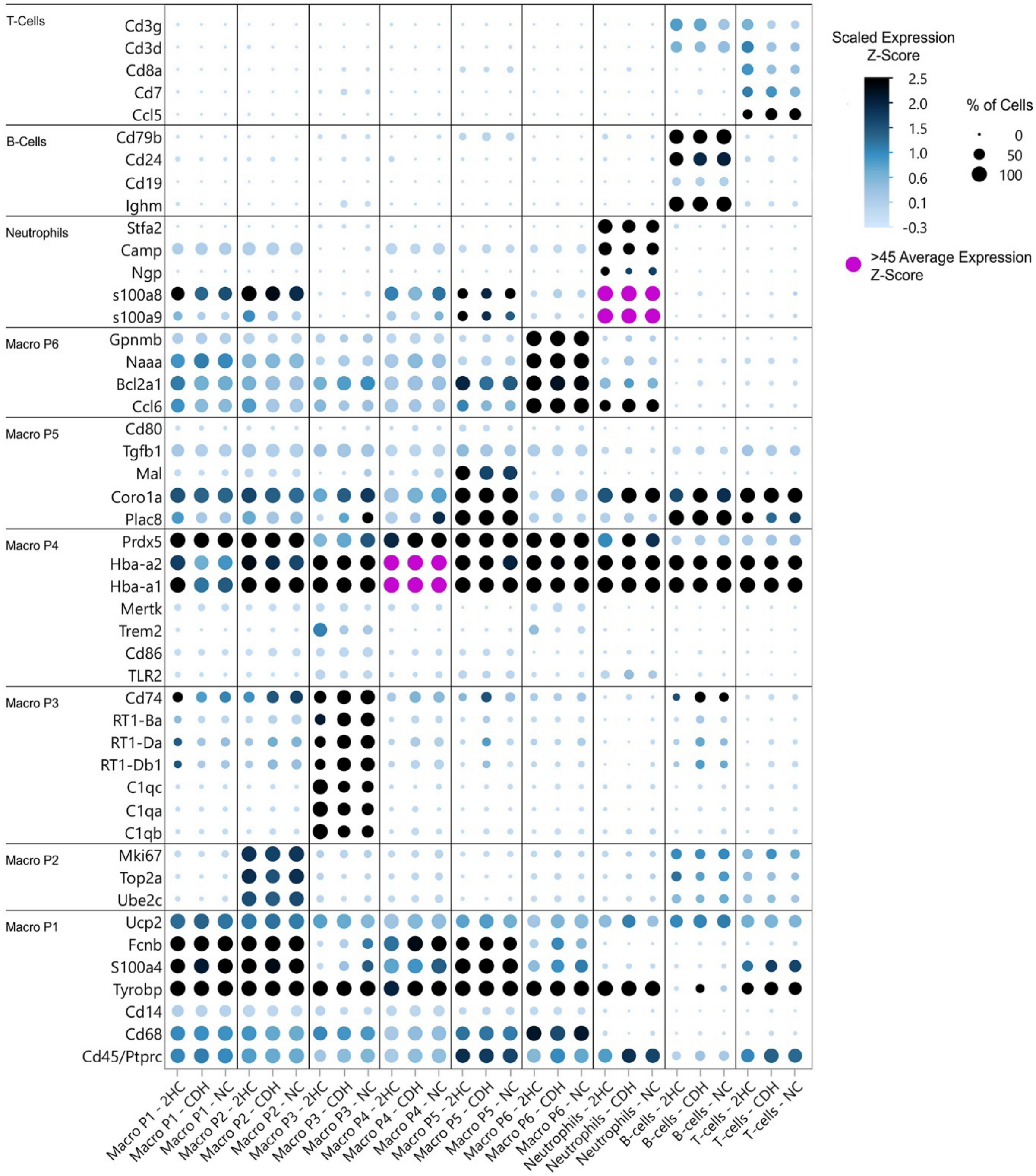

Supplemental Figure S3. Known cell type markers identified discrete immune cell clusters within each experimental group.

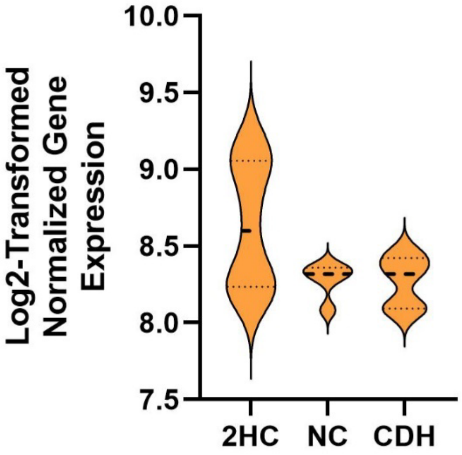

Supplemental Figure S4. AT1 *Vegfa* expression changes did not account for reductions in *Ca4*+ microvascular endothelial cells. No significant differences in type I alveolar cell *Vegfa* expression were observed between experimental groups.

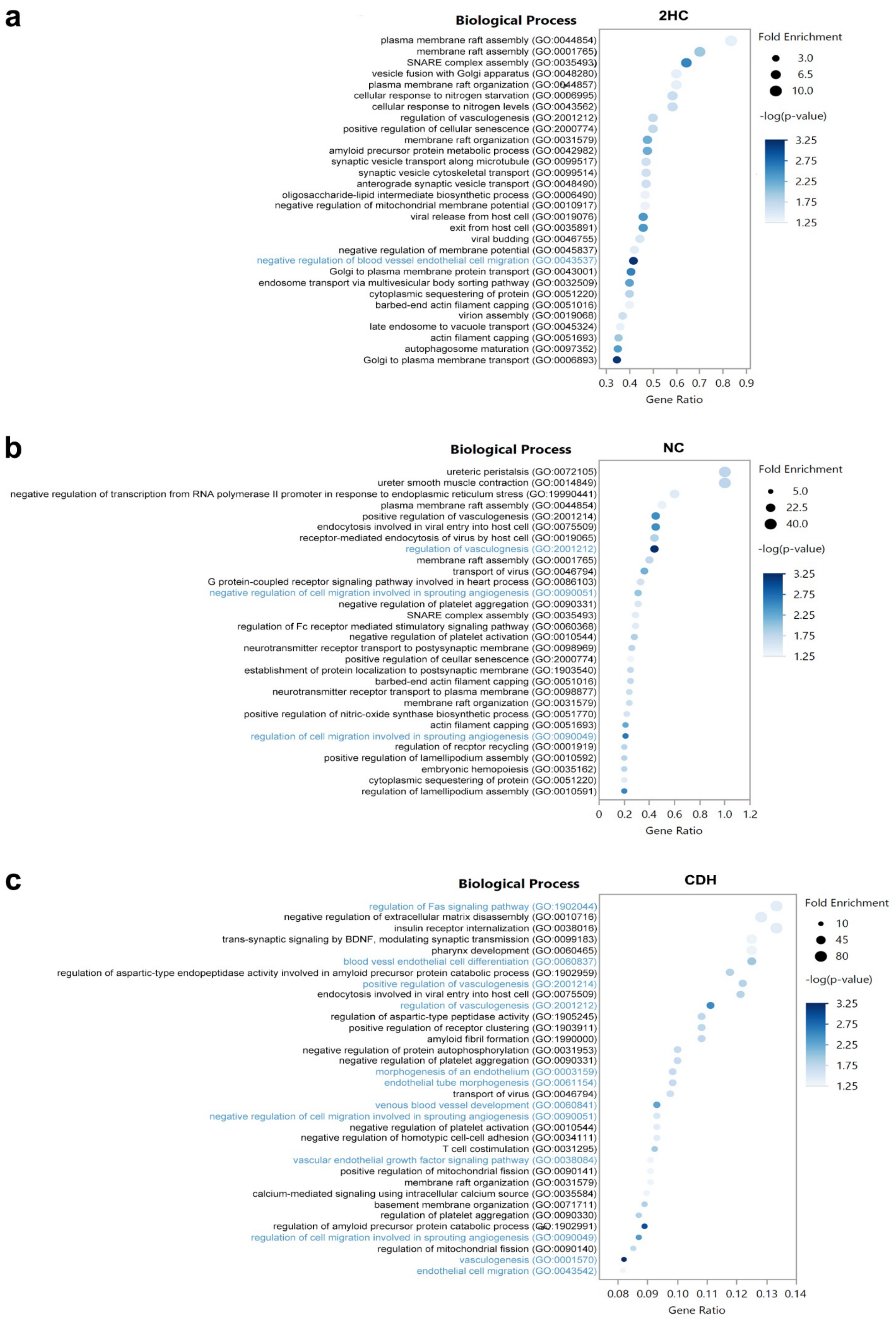
a
Biological Process
2HC
plasma membrane raft assembly (GO:0044854)
membrane raft assembly (GO:0001765)
SNARE complex assembly (GO:0035493)
vesicle fusion with Golgi apparatus (GO:0048280)
plasma membrane raft organization (GO:0044857)
cellular response to nitrogen starvation (GO:0006995)
cellular response to nitrogen levels (GO:0043562)
regulation of vasculogenesis (GO:2001212)
positive regulation of cellular senescence (GO:2000774)
membrane raft organization (GO:0031579)
amyloid precursor protein metabolic process (GO:0042982)
synaptic vesicle transport along microtubule (GO:0099517)
synaptic vesicle cytoskeletal transport (GO:0099514)
anterograde synaptic vesicle transport (GO:0048490)
oligosaccharide-lipid intermediate biosynthetic process (GO:0006490)
negative regulation of mitochondrial membrane potential (GO:0010917)
viral release from host cell (GO:0019076)
exit from host cell (GO:0035891)
viral budding (GO:0046755)
negative regulation of membrane potential (GO:0045837)
negative regulation of blood vessel endothelial cell migration (GO:0043537)
Golgi to plasma membrane protein transport (GO:0043001)
endosome transport via multivesicular body sorting pathway (GO:0032509)
cytoplasmic sequestering of protein (GO:0051220)
barbed-end actin filament capping (GO:0051016)
virion assembly (GO:0019068)
late endosome to vacuole transport (GO:0045324)
actin filament capping (GO:0051693)
autophagosome maturation (GO:0097352)
Golgi to plasma membrane transport (GO:0006893)
0.3 0.4 0.5 0.6 0.7 0.8 0.9
Gene Ratio
Fold Enrichment
3.0
6.5
10.0
-log(p-value)
3.25
2.75
2.25
1.75
1.25
b
Biological Process
NC
ureteric peristalsis (GO:0072105)
ureter smooth muscle contraction (GO:0014849)
negative regulation of transcription from RNA polymerase II promoter in response to endoplasmic reticulum stress (GO:19990441)
plasma membrane raft assembly (GO:0044854)
positive regulation of vasculogenesis (GO:2001214)
endocytosis involved in viral entry into host cell (GO:0075509)
receptor-mediated endocytosis of virus by host cell (GO:0019065)
regulation of vasculogenesis (GO:2001212)
membrane raft assembly (GO:0001765)
transport of virus (GO:0046794)
G protein-coupled receptor signaling pathway involved in heart process (GO:0086103)
negative regulation of cell migration involved in sprouting angiogenesis (GO:0090051)
negative regulation of platelet aggregation (GO:0090331)
SNARE complex assembly (GO:0035493)
regulation of Fc receptor mediated stimulatory signaling pathway (GO:0060368)
negative regulation of platelet activation (GO:0010544)
neurotransmitter receptor transport to postsynaptic membrane (GO:0098969)
positive regulation of ceullar senescence (GO:2000774)
establishment of protein localization to postsynaptic membrane (GO:1903540)
barbed-end actin filament capping (GO:0051016)
neurotransmitter receptor transport to plasma membrane (GO:0098877)
membrane raft organization (GO:0031579)
positive regulation of nitric-oxide synthase biosynthetic process (GO:0051770)
actin filament capping (GO:0051693)
regulation of cell migration involved in sprouting angiogenesis (GO:0090049)
regulation of recptor recycling (GO:0001919)
positive regulation of lamellipodium assembly (GO:0010592)
embryonic hemopoiesis (GO:0035162)
cytoplasmic sequestering of protein (GO:0051220)
regulation of lamellipodium assembly (GO:0010591)
0 0.2 0.4 0.6 0.8 1.0 1.2
Gene Ratio
Fold Enrichment
5.0
22.5
40.0
-log(p-value)
3.25
2.75
2.25
1.75
1.25
c
Biological Process
CDH
regulation of Fas signaling pathway (GO:1902044)
negative regulation of extracellular matrix disassembly (GO:0010716)
insulin receptor internalization (GO:0038016)
trans-synaptic signaling by BDNF, modulating synaptic transmission (GO:0099183)
pharynx development (GO:0060465)
blood vessl endothelial cell differentiation (GO:0060837)
regulation of aspartic-type endopeptidase activity involved in amyloid precursor protein catabolic process (GO:1902959)
positive regulation of vasculogenesis (GO:2001214)
endocytosis involved in viral entry into host cell (GO:0075509)
regulation of vasculogenesis (GO:2001212)
regulation of aspartic-type peptidase activity (GO:1905245)
positive regulation of receptor clustering (GO:1903911)
amyloid fibril formation (GO:1990000)
negative regulation of protein autophosphorylation (GO:0031953)
negative regulation of platelet aggregation (GO:0090331)
morphogenesis of an endothelium (GO:0003159)
endothelial tube morphogenesis (GO:0061154)
transport of virus (GO:0046794)
venous blood vessel development (GO:0060841)
negative regulation of cell migration involved in sprouting angiogenesis (GO:0090051)
negative regulation of platelet activation (GO:0010544)
negative regulation of homotypic cell-cell adhesion (GO:0034111)
T cell costimulation (GO:0031295)
vascular endothelial growth factor signaling pathway (GO:0038084)
positive regulation of mitochondrial fission (GO:0090141)
membrane raft organization (GO:0031579)
calcium-mediated signaling using intracellular calcium source (GO:0035584)
basement membrane organization (GO:0071711)
regulation of platelet aggregation (GO:0090330)
regulation of amyloid precursor protein catabolic process (GO:1902991)
regulation of cell migration involved in sprouting angiogenesis (GO:0090049)
regulation of mitochondrial fission (GO:0090140)
vasculogenesis (GO:0001570)
endothelial cell migration (GO:0043542)
0.08 0.09 0.10 0.11 0.12 0.13 0.14
Gene Ratio
Fold Enrichment
10
45
80
-log(p-value)
3.25
2.75
2.25
1.75
1.25

Supplemental Figure S5. Gene ontology analysis revealed increased enrichment for vascular development and endothelial migration in CDH mvCa4+ endothelial cells. These analyses represent gene enrichment in mvCa4+ endothelial cells compared to all other endothelial cells within the 2HC (**a**), NC (**b**) and CDH (**c**) experimental groups. The top 30 biological process results from PANTHER gene ontology analysis are shown, plus four additional, relevant processes from the next 25 biological processes are shown for the CDH group. Biological processes highlighted in red are relevant to blood vessel formation and endothelial cell function. The -log(p-value) is a transformation of the false discovery rate (FDR) adjusted p-value. Note, that the scale for the fold enrichment varies between the three groups, such that there are both more angiogenic biological processes and significantly higher fold enrichment for the CDH mvCa4+ ECs.

Supplemental Table S1. 2HC mvEC PANTHER Gene Ontology Analysis

Biological Process	Fold Change	Gene Ratio	FDR Adjusted *P*-Value
electron transport coupled proton transport (GO:0015990)	32.06	0.60	2.35E-02
negative regulation of ubiquitin protein ligase activity (GO:1904667)	28.77	0.54	1.57E-05
energy coupled proton transmembrane transport, against electrochemical gradient (GO:0015988)	22.90	0.43	4.08E-02
muscle filament sliding (GO:0030049)	22.90	0.43	4.06E-02
venous blood vessel development (GO:0060841)	18.70	0.35	9.72E-05
negative regulation of ubiquitin-protein transferase activity (GO:0051444)	15.58	0.29	2.37E-04
regulation of ubiquitin protein ligase activity (GO:1904666)	14.38	0.27	3.47E-04
regulation of cell-cell adhesion mediated by cadherin (GO:2000047)	12.57	0.24	3.45E-02
basement membrane organization (GO:0071711)	11.05	0.21	4.69E-03
inner mitochondrial membrane organization (GO:0007007)	10.34	0.19	5.80E-03
positive regulation of signal transduction by p53 class mediator (GO:1901798)	10.02	0.19	6.56E-03
vascular endothelial growth factor receptor signaling pathway (GO:0048010)	9.89	0.19	2.07E-02
ribosomal large subunit assembly (GO:0000027)	8.22	0.15	1.38E-02
response to thyroid hormone (GO:0097066)	8.13	0.15	5.56E-03
vasculogenesis (GO:0001570)	8.01	0.15	3.58E-05
negative regulation of ubiquitin-dependent protein catabolic process (GO:2000059)	7.92	0.15	2.38E-03
regulation of actin cytoskeleton reorganization (GO:2000249)	7.63	0.14	1.85E-02
establishment of endothelial barrier (GO:0061028)	7.63	0.14	4.61E-02
cellular response to vascular endothelial growth factor stimulus (GO:0035924)	7.46	0.14	2.04E-02
ribosome assembly (GO:0042255)	7.39	0.14	2.77E-05
positive regulation of G1/S transition of mitotic cell cycle (GO:1900087)	6.97	0.13	2.60E-02
regulation of ubiquitin-protein transferase activity (GO:0051438)	6.68	0.13	1.32E-02
aerobic electron transport chain (GO:0019646)	6.48	0.12	6.59E-03
regulation of extracellular matrix organization (GO:1903053)	6.41	0.12	3.58E-02
ATP synthesis coupled electron transport (GO:0042773)	6.33	0.12	3.54E-03
positive regulation of DNA-templated transcription initiation (GO:2000144)	6.29	0.12	3.82E-02
liver regeneration (GO:0097421)	6.24	0.12	3.82E-03
negative regulation of proteolysis involved in protein catabolic process (GO:1903051)	6.19	0.12	8.33E-03
endothelial cell differentiation (GO:0045446)	6.00	0.11	1.99E-03
mitochondrial ATP synthesis coupled electron transport (GO:0042775)	5.78	0.11	1.18E-02
oxidative phosphorylation (GO:0006119)*	5.60	0.10	1.46E-03
endothelium development (GO:0003158)*	5.14	0.10	5.59E-03
respiratory electron transport chain (GO:0022904)*	4.86	0.09	1.43E-02
aerobic respiration (GO:0009060)*	4.82	0.09	1.07E-03
positive regulation of endothelial cell proliferation (GO:0001938)*	4.76	0.09	1.57E-02
positive regulation of angiogenesis (GO:0045766)*	4.48	0.08	5.63E-04
positive regulation of vasculature development (GO:1904018)*	4.48	0.08	5.57E-04

*Above the black line are the top 30 biological processes, as sorted by fold enrichment. The biological processes below the heavy black line are relevant processes selected from the next 30 top results.

Supplemental Table S2. NC mvEC PANTHER Gene Ontology Analysis			
**Biological Process**	**Fold Change**	**Gene Ratio**	**FDR Adjusted *P*-Value**
apelin receptor signaling pathway (GO:0060183)	95.93	0.67	2.32E-02
negative regulation of adenylate cyclase-activating adrenergic receptor signaling pathway involved in heart process (GO:0140199)	95.93	0.67	2.31E-02
melanocyte migration (GO:0097324)	95.93	0.67	2.31E-02
electron transport coupled proton transport (GO:0015990)	86.34	0.60	1.82E-03
protein localization to bicellular tight junction (GO:1902396)	71.95	0.50	2.52E-03
caveola assembly (GO:0070836)	71.95	0.50	3.11E-02
regulation of adenylate cyclase-activating adrenergic receptor signaling pathway involved in heart process (GO:0140192)	71.95	0.50	3.10E-02
positive regulation of mast cell proliferation (GO:0070668)	71.95	0.50	2.50E-03
trans-synaptic signaling by BDNF, modulating synaptic transmission (GO:0099183)	71.95	0.50	3.09E-02
energy coupled proton transmembrane transport, against electrochemical gradient (GO:0015988)	61.67	0.43	3.24E-03
negative regulation of adenylate cyclase-activating adrenergic receptor signaling pathway (GO:0071878)	57.56	0.40	4.01E-02
negative regulation of protein neddylation (GO:2000435)	57.56	0.40	4.00E-02
positive regulation of endothelial cell chemotaxis by VEGF-activated vascular endothelial growth factor receptor signaling pathway (GO:0038033)	57.56	0.40	3.99E-02
trans-synaptic signaling by BDNF (GO:0099191)	57.56	0.40	3.98E-02
dichotomous subdivision of terminal units involved in salivary gland branching (GO:0060666)	57.56	0.40	3.97E-02
plasma membrane raft assembly (GO:0044854)	47.97	0.33	4.87E-02
mast cell proliferation (GO:0070662)	47.97	0.33	4.86E-02
positive regulation of gap junction assembly (GO:1903598)	47.97	0.33	4.85E-02
regulation of gap junction assembly (GO:1903596)	47.97	0.33	5.21E-03
positive regulation of establishment of endothelial barrier (GO:1903142)	43.17	0.30	6.45E-03
positive regulation of endothelial cell development (GO:1901552)	43.17	0.30	6.42E-03
regulation of mast cell proliferation (GO:0070666)	43.17	0.30	6.39E-03
positive regulation of vasculogenesis (GO:2001214)	39.25	0.27	7.74E-03
venous blood vessel development (GO:0060841)	35.98	0.25	1.30E-04
retina vasculature morphogenesis in camera-type eye (GO:0061299)	30.84	0.21	1.21E-02
angiogenesis involved in wound healing (GO:0060055)	28.78	0.20	1.40E-02
negative regulation of vasoconstriction (GO:0045906)	28.78	0.20	1.40E-02
regulation of vasculogenesis (GO:2001212)	26.98	0.19	1.60E-02
negative regulation of vascular permeability (GO:0043116)	26.16	0.18	2.71E-03
retina vasculature development in camera-type eye (GO:0061298)	25.03	0.17	3.01E-03
establishment of endothelial barrier (GO:0061028)*	24.67	0.17	7.35E-05
regulation of establishment of endothelial barrier (GO:1903140)*	20.56	0.14	2.78E-02
vascular endothelial growth factor signaling pathway (GO:0038084)*	20.56	0.14	2.77E-02
regulation of endothelial cell development (GO:1901550)*	20.56	0.14	2.76E-02
positive regulation of cell migration involved in sprouting angiogenesis (GO:0090050)*	20.56	0.14	2.75E-02
regulation of vascular permeability (GO:0043114)*	17.62	0.12	3.19E-04
endothelial cell development (GO:0001885)*	17.07	0.12	7.42E-05
vasculogenesis (GO:0001570)*	16.19	0.11	3.92E-06
vascular endothelial growth factor receptor signaling pathway (GO:0048010)*	15.99	0.11	4.47E-02
branching involved in blood vessel morphogenesis (GO:0001569)*	15.56	0.11	1.09E-02

aerobic electron transport chain (GO:0019646)*	15.26	0.11	1.33E-04
endothelial cell differentiation (GO:0045446)*	14.55	0.10	8.29E-06
*Above the black line are the top 30 biological processes, as sorted by fold enrichment. The biological processes below the heavy black line are relevant processes selected from the next 30 top results.			

Supplemental Table S3. CDH mvEC PANTHER Gene Ontology Analysis			
**Biological Process**	**Fold Change**	**Gene Ratio**	**FDR Adjusted *P*-Value**
negative regulation of adenylate cyclase-activating adrenergic receptor signaling pathway involved in heart process (GO:0140199)	> 100	0.67	1.58E-02
regulation of adenylate cyclase-activating adrenergic receptor signaling pathway involved in heart process (GO:0140192)	> 100	0.50	2.09E-02
caveola assembly (GO:0070836)	> 100	0.50	2.10E-02
dichotomous subdivision of terminal units involved in salivary gland branching (GO:0060666)	83.83	0.40	2.63E-02
electron transport coupled proton transport (GO:0015990)	83.83	0.40	2.64E-02
positive regulation of endothelial cell chemotaxis by VEGF-activated vascular endothelial growth factor receptor signaling pathway (GO:0038033)	83.83	0.40	2.65E-02
negative regulation of adenylate cyclase-activating adrenergic receptor signaling pathway (GO:0071878)	83.83	0.40	2.66E-02
positive regulation of mast cell proliferation (GO:0070668)	69.85	0.33	3.22E-02
protein localization to bicellular tight junction (GO:1902396)	69.85	0.33	3.24E-02
plasma membrane raft assembly (GO:0044854)	69.85	0.33	3.25E-02
energy coupled proton transmembrane transport, against electrochemical gradient (GO:0015988)	59.88	0.29	3.91E-02
regulation of matrix metallopeptidase secretion (GO:1904464)	52.39	0.25	4.66E-02
positive regulation of cell migration by vascular endothelial growth factor signaling pathway (GO:0038089)	52.39	0.25	4.67E-02
positive regulation of early endosome to late endosome transport (GO:2000643)	52.39	0.25	4.69E-02
retina vasculature morphogenesis in camera-type eye (GO:0061299)	44.91	0.21	6.70E-03
venous blood vessel development (GO:0060841)	41.91	0.20	1.02E-03
negative regulation of vascular permeability (GO:0043116)	38.10	0.18	1.25E-03
maintenance of blood-brain barrier (GO:0035633)	34.93	0.17	1.08E-02
positive regulation of cell migration involved in sprouting angiogenesis (GO:0090050)	29.94	0.14	1.47E-02
vascular endothelial growth factor signaling pathway (GO:0038084)	29.94	0.14	1.48E-02
basement membrane organization (GO:0071711)	28.91	0.14	2.58E-03
embryonic hemopoiesis (GO:0035162)	27.94	0.13	2.83E-03
retina vasculature development in camera-type eye (GO:0061298)	27.33	0.13	1.72E-02
antigen processing and presentation of peptide antigen via MHC class Ib (GO:0002428)	27.04	0.13	3.03E-03
cellular response to thyroid hormone stimulus (GO:0097067)	26.20	0.13	1.87E-02
mitochondrial electron transport, cytochrome c to oxygen (GO:0006123)	26.20	0.13	1.88E-02
antigen processing and presentation via MHC class Ib (GO:0002475)	24.65	0.12	3.89E-03
establishment of endothelial barrier (GO:0061028)	23.95	0.11	4.22E-03
antigen processing and presentation of endogenous peptide antigen via MHC class I (GO:0019885)	23.95	0.11	4.25E-03
vascular endothelial growth factor receptor signaling pathway (GO:0048010)	23.28	0.11	2.33E-02
branching involved in blood vessel morphogenesis (GO:0001569)*	22.66	0.11	4.89E-03
antigen processing and presentation of endogenous peptide antigen (GO:0002483)*	22.66	0.11	4.93E-03
antigen processing and presentation of endogenous peptide antigen via MHC class I via ER pathway, TAP-independent (GO:0002486)*	21.68	0.10	2.64E-02
antigen processing and presentation of endogenous antigen (GO:0019883)*	21.49	0.10	5.52E-03
regulation of vascular permeability (GO:0043114)*	21.38	0.10	1.16E-03

vasculogenesis (GO:0001570)*	20.96	0.10	6.12E-06
antigen processing and presentation of endogenous peptide antigen via MHC class Ib (GO:0002476)*	20.96	0.10	2.84E-02
antigen processing and presentation of endogenous peptide antigen via MHC class I via ER pathway (GO:0002484)*	20.96	0.10	2.85E-02
negative regulation of endothelial cell apoptotic process (GO:2000352)*	19.65	0.09	3.23E-02
cellular response to vascular endothelial growth factor stimulus (GO:0035924)*	19.49	0.09	7.01E-03
antigen processing and presentation of peptide antigen via MHC class I (GO:0002474)*	17.84	0.09	8.98E-03
positive regulation of blood vessel endothelial cell migration (GO:0043536)*	17.76	0.08	2.13E-03
endothelial cell development (GO:0001885)*	17.76	0.08	2.15E-03
negative regulation of endothelial cell proliferation (GO:0001937)*	17.46	0.08	9.48E-03
mast cell activation (GO:0045576)*	17.46	0.08	4.14E-02
positive regulation of T cell mediated cytotoxicity (GO:0001916)*	17.11	0.08	9.95E-03
regulation of cell migration involved in sprouting angiogenesis (GO:0090049)*	16.12	0.08	4.93E-02
regulation of endothelial cell apoptotic process (GO:2000351)*	15.24	0.07	1.37E-02
regulation of blood vessel endothelial cell migration (GO:0043535)*	15.12	0.07	2.19E-04
regulation of T cell mediated cytotoxicity (GO:0001914)*	14.45	0.07	1.56E-02
endothelial cell differentiation (GO:0045446)*	14.13	0.07	1.19E-03
ATP synthesis coupled electron transport (GO:0042773)*	13.79	0.07	4.93E-03

*Above the black line are the top 30 biological processes, as sorted by fold enrichment. The biological processes below the heavy black line are relevant processes selected from the next 30 top results.

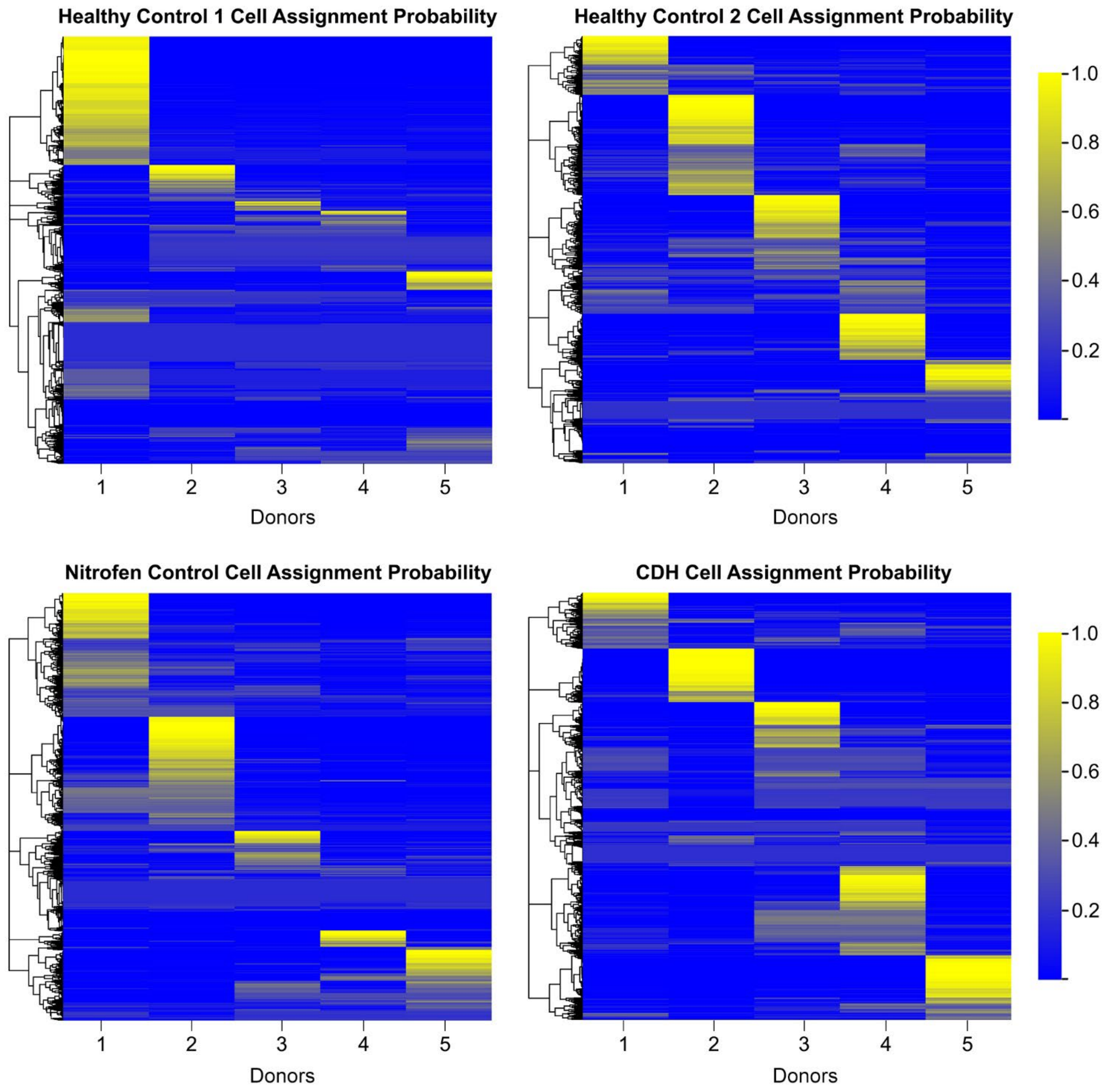

Supplemental Figure S6. Heatmaps of donor cell assignment probabilities determined by Vireo germline genotype-based demultiplexing. Unsupervised hierarchical clustering was applied to rows (cells) and plotted using the pheatmap function in R (version 4.1.1). Per-cell assignments were based on germline genotype patterns representative of distinct rat samples. Cells with equal donor probabilities were randomly assigned to donors.
